# Enhanced H_2_O_2_ Production via Photocatalytic O_2_ Reduction over Structurally-Modified Poly(heptazine imide)

**DOI:** 10.1021/acs.chemmater.2c00528

**Published:** 2022-06-08

**Authors:** Pankaj Sharma, Thomas J. A. Slater, Monika Sharma, Michael Bowker, C. Richard A. Catlow

**Affiliations:** † Cardiff Catalysis Institute, School of Chemistry, Cardiff University, Cardiff CF10 3AT, United Kingdom; ‡ UK Catalysis Hub, Research Complex at Harwell, Rutherford Appleton Laboratory, Harwell OX11 0FA, United Kingdom; § Department of Chemistry, Kurukshetra University, Kurukshetra 136 119, Haryana, India; ∥ Department of Chemistry, University College London, 20 Gordon Street, London WC1 HOAJ, United Kingdom

## Abstract

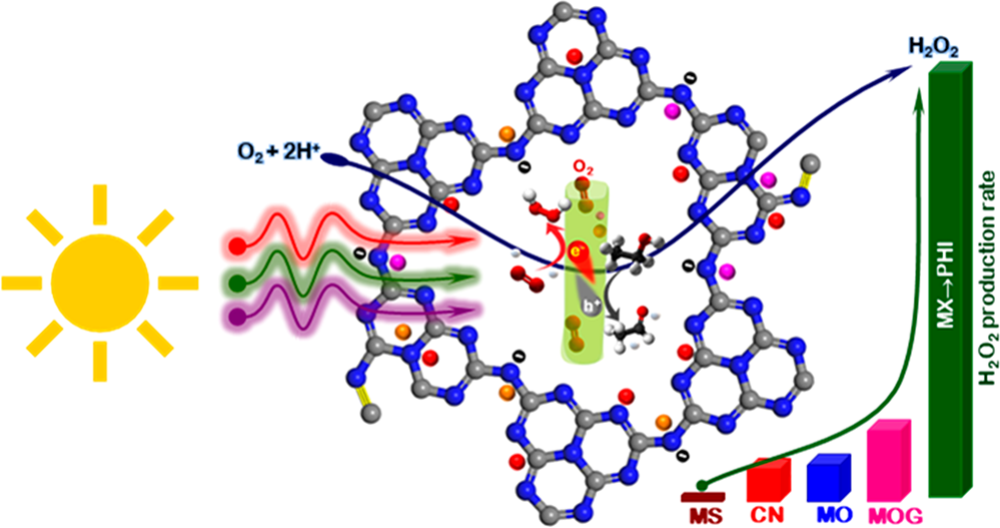

Solar H_2_O_2_ produced by O_2_ reduction provides a green, efficient, and ecological alternative to the industrial anthraquinone process and H_2_/O_2_ direct-synthesis. We report efficient photocatalytic H_2_O_2_ production at a rate of 73.4 mM h^–1^ in the presence of a sacrificial donor on a structurally engineered catalyst, alkali metal-halide modulated poly(heptazine imide) (MX → PHI). The reported H_2_O_2_ production is nearly 150 and >4250 times higher than triazine structured pristine carbon nitride under UV–visible and visible light (≥400 nm) irradiation, respectively. Furthermore, the solar H_2_O_2_ production rate on MX → PHI is higher than most of the previously reported carbon nitride (triazine, tri-s-triazine), metal oxides, metal sulfides, and other metal–organic photocatalysts. A record high AQY of 96% at 365 nm and 21% at 450 nm was observed. We find that structural modulation by alkali metal-halides results in a highly photoactive MX → PHI catalyst which has a broader light absorption range, enhanced light absorption ability, tailored bandgap, and a tunable band edge position. Moreover, this material has a different polymeric structure, high O_2_ trapping ability, interlayer intercalation, as well as surface decoration of alkali metals. The specific C≡N groups and surface defects, generated by intercalated MX, were also considered as potential contributors to the separation of photoinduced electron–hole pairs, leading to enhanced photocatalytic activity. A synergy of all these factors contributes to a higher H_2_O_2_ production rate. Spectroscopic data help us to rationalize the exceptional photochemical performance and structural characteristics of MX → PHI.

## 1 Introduction

As a consequence of the growing demand for clean energy,^1−3^ there has been extensive research in recent decades aimed at replacing conventional fuels with carbon-free energy sources.^4−6^ A higher energy density H_2_O_2_ (3.0 MJ L^–1^ 60% H_2_O_2_) has been projected as a potential energy carrier that is relatively free from storage and transport issues.^7^ Furthermore, H_2_O_2_ already has significant importance in medical and industrial uses. The estimated global market for H_2_O_2_ was 4.5 million metric tons in 2020, and it is projected to reach 5.7 million metric tons by 2027.^8^

Despite its importance as a chemical and ecological oxidant, the production of H_2_O_2_ still relies mainly on the energy intensive and waste generating industrial anthraquinone auto-oxidation process.^9^ An alternative approach for H_2_O_2_ is the direct electrolysis of H_2_/O_2_ over expensive metal catalysts.^10^ However, its dependence on H_2_, i.e., the consumption of one carbon-free energy source (H_2_) to produce another carbon-free energy source (H_2_O_2_), shows that this process cannot at present be regarded as a suitable alternative. Due to the drawbacks of direct electrolysis, the photocatalytic H_2_O_2_ production via direct or indirect utilization of solar power has generated substantial interest as a potential, sustainable route.^11,12^ However, even with significant advances in metallic and nonmetallic photocatalysts (PCs), existing photocatalytic systems can only generate low yields of H_2_O_2_.^13−19^

Among nonmetallic photocatalysts, carbon–nitrogen (C–N)-based materials have attracted much attention, as they have a suitable conduction-band edge to carry out the two-electron transfer photochemical O_2_ reduction reactions (PCORR) for H_2_O_2_ generation. It is also possible easily to improve their catalytic efficacy by simple structural modifications, where various strategies including alkali metal doping,^16,20,21^ cocatalyst loading,^22^ structural/heterostructural engineering,^23−25^ band alignment,^26^ structural defects/vacancy center creation,^27−29^ and surface shielding^15,30,31^ have been adopted. Hirai and co-workers reported the synthesis of a metal-free pyromellitic diimide-doped carbon nitride (g-C_3_N_4_/PDI) photocatalyst hybridized with reduced graphene oxide (rGO) for photochemical production of H_2_O_2_. They successfully generated nearly 20 mM H_2_O_2_ by O_2_ reduction in 90% (v) 2-propanol/water using a 1.7 g L^–1^ photocatalyst suspension for 9 h irradiation,^24^ which is the highest reported solar H_2_O_2_ production yet obtained via O_2_ reduction in the presence of a sacrificial agent. Recently, Quan et al.^16^ reported that the synergistic effect of Na^+^, K^+^ dopants and N vacancies on C_3_N_4_ resulted in a H_2_O_2_ production rate of 10.2 mM h^–1^, which is 89.5-fold higher than that of pristine C_3_N_4_. Unfortunately, despite the extensive efforts toward a polymeric structured g-C_3_N_4_ (triazine → tri-s-triazine) synthesis, including doping and defect and structural engineering, the highest values obtained for H_2_O_2_ production on the g-C_3_N_4_ based photocatalysts such as g-C_3_N_4_/PDI/rGO^24^ and Na^+^, K^+^/N@g-C_3_N_4_^16^ are similar to those reported earlier for metal oxides^15,32,33^ sulfides,^34,35^ and molecular^14,36^ photocatalysts. Indeed, in all reported studies, the main obstacle for developing solar-driven H_2_O_2_ production as a suitable alternative is the low yield of H_2_O_2_ generated.

There is, therefore, a pressing need for the development of an effective photocatalyst that could greatly increase photocatalytic H_2_O_2_ production. Stimulated by the earlier reported strategies, we have synthesized a new photocatalyst that combines properties including a higher intrinsic surface area, modified electronic structure, reduced band gap, and defect sites for enhanced H_2_O_2_ production. To this end, we have successfully synthesized an alkali metal-halide (MX, M = K^+^; Li^+^, X = Cl^–^) modulated C–N based poly(heptazine imide) (PHI) molecular photocatalyst, MX → PHI for PCORR to produce a much higher yield of H_2_O_2_ (73.4 mM h^–1^) than obtained previously. The present structurally modulated MX → PHI photocatalyst was synthesized by facile polymerization of an environmentally benign precursor, urea, in the presence of alkali metal halides. A combination of microscopic, spectroscopic, and optoelectronic techniques verified the successful intercalation of MXs, found that the 3D-hollow fibers had a lamellar structure, and verified a broadening of the light absorption range and an enhanced light absorption ability of the synthesized catalyst, leading to substantially increased H_2_O_2_ production rates. Our work clearly demonstrates the potential of MX → PHI for PCORR generating high yields of H_2_O_2_.

## 2 Results and Discussion

### 2.1 MX → PHI Growth and Characteristics Evaluation

**Figure 1:**
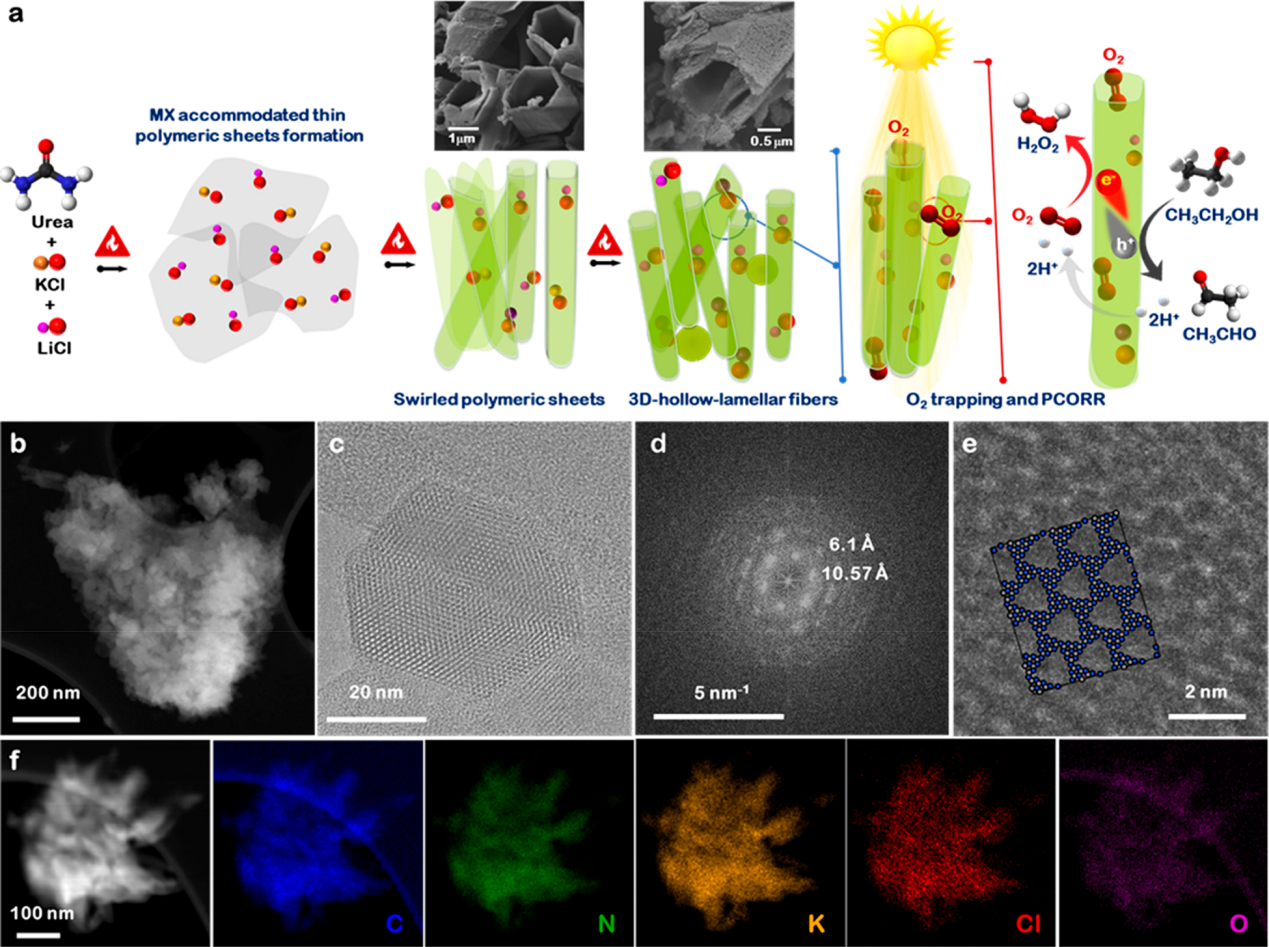
Alkali metal halide incorporated lamellar fiber structured PHI molecular photocatalyst. (a) Schematic illustration highlighting the growth mechanism of hollow fiber MX → PHI particles/rods. The conceptual graphic shows the deep penetration of solar light, light radiation trapping, and O_2_ gas molecule absorbance/confinement resulting in photochemical performance enhancement. (b) HAADF-STEM image of agglomerated MX → PHI. (c) HRTEM image of individual MX → PHI particles with associated fast Fourier transform (FFT) (d). (e) HRTEM image of MX → PHI particles with superimposed structure (C, N, and embedded alkali metal halides atoms colored in gray, blue, and red, respectively). (f) HAADF-STEM image and corresponding XEDS maps for carbon, nitrogen, potassium, chlorine, and oxygen. All elements appear to be distributed homogeneously within an agglomerated region.

Solid-state polymerization of tri-s-triazine structured metal-doped g-C_3_N_4_ typically involves two steps: thermal condensation of nitrogen-rich precursors (urea, melamine, etc.) followed by ionothermal polymerization of the C–N based polymer.^37^ The present highly photoactive MX → PHI photocatalyst was, however, directly synthesized from urea using a single-step ionothermal polymerization. To analyze the growth of the MX → PHI photocatalyst, two separate sets of experiments were performed. The samples collected at 350 and 500 °C during ionothermal polymerization of urea were denoted as MX → PHI_350_ and MX → PHI_500_, respectively, and the growth process is illustrated schematically in Figure 1a.

The morphologies of MX → PHI_350_, MX → PHI_500_, and MX → PHI (the final product after 5 h polymerization at 550 °C) were characterized by scanning electron microscopy (SEM) to substantiate the growth mechanism (Figures S1–S6). The SEM micrographs of solidified MX → PHI_350_ (Figure S1) revealed coiling of thin polymeric sheets of basic carbon nitride (BCN) to form swirled polymeric hollow fibers/rods at the initial stage of polymerization, which later transformed into highly crystalline hollow fibers/rods (Figures S3 and S5). Furthermore, the presence of alkali metal-halide ions results in the polymeric sheets folding to achieve energetically favorable hollow fibers, with a self-shaping crystal growth mechanism.^38,39^ MX → PHI therefore has a 3D-hollow fiber morphology which consists of macroporous lamellar walls with a higher thickness compared to triazine structured BCN, which has an aggregated sheetlike morphology (Figures S7 and S8).

High-angle annular dark field (HAADF) scanning transmission electron microscope (STEM) images (Figure 1b) revealed that the MX → PHI materials have aggregates of nanosized particles and rods. High-resolution TEM images of an MX → PHI particle (Figure 1c,d, and Figure S9) confirm the crystalline nature of MX → PHI. Characteristic distances of 10.57 Å, corresponding to the (110) plane in poly(heptazine imide), and 3.25 Å, corresponding to the (001) plane were found. The poly(heptazine imide) structure can be matched to features in the HRTEM images (Figure 1e) and the Fourier transform of a simulated HRTEM image contains low-frequency peaks that match those in the Fourier transform of the experimental images (Figure S10).

The insertion of alkali metal halides was confirmed by X-ray energy dispersive spectroscopy (XEDS) in the STEM. The HAADF-STEM image and corresponding XEDS maps of individual elements (carbon, nitrogen, oxygen, potassium, and chlorine) (Figure 1f) reveal successful and uniform distributions of each element into MX → PHI, which is consistent with the SEM-XEDS data (Figures S2, S4, and S6). The homogeneous distribution of K and Cl throughout the sample demonstrates that ionothermal polymerization of urea results in the diffusion of the alkali metal halide into the growing polymeric unit of the heptazine imide.

**Figure 2:**
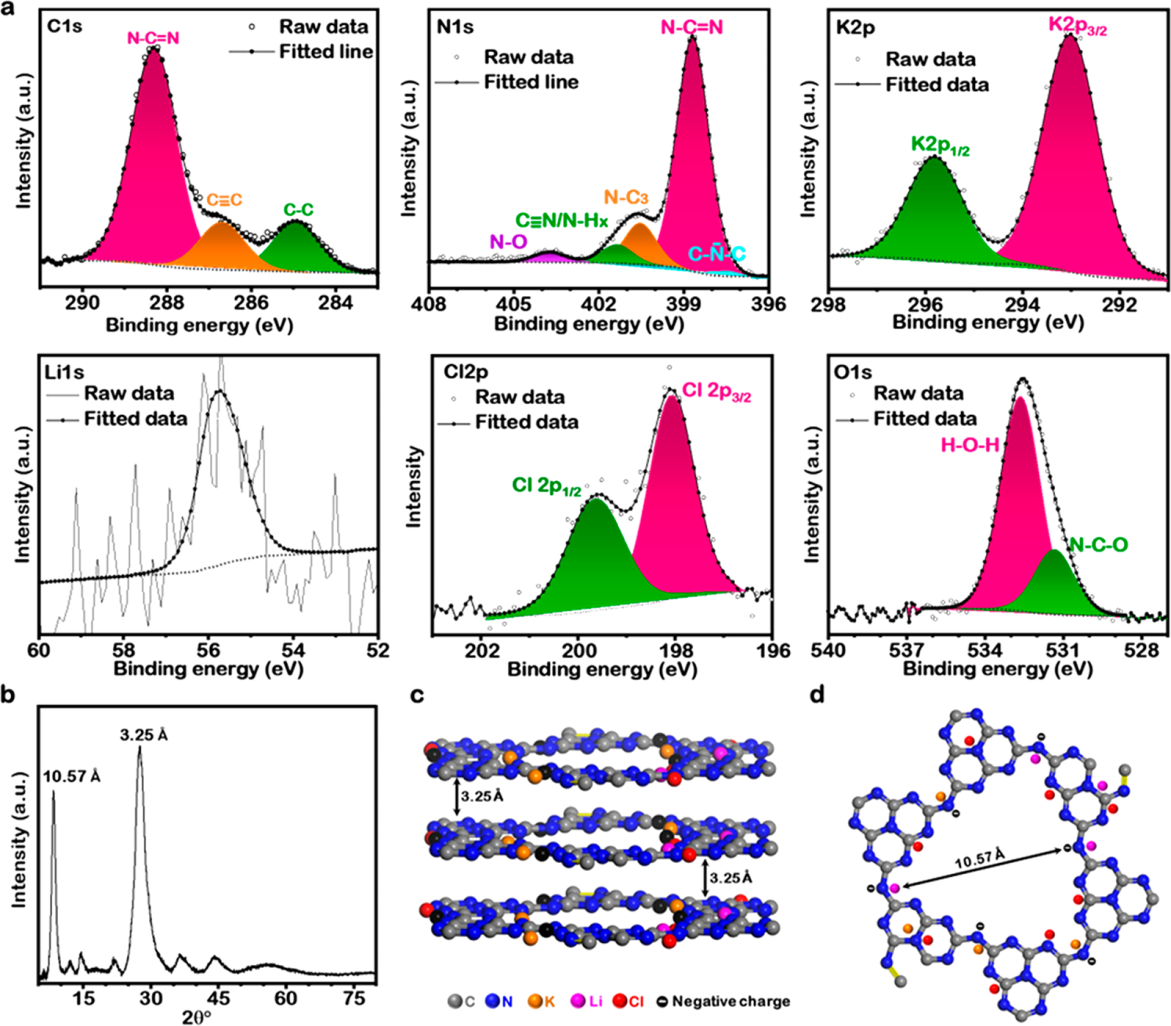
Surface and core structure evaluation of MX → PHI. (a) Core-level XPS spectra for C 1s, N 1s, K 2p, Li 1s, Cl 2p, and O 1s present in MX → PHI. (b) Powder XRD pattern of the layered MX → PHI. (c,d) Layered and in plane structure of MX → PHI.

X-ray photoelectron spectroscopy (XPS) was used to characterize the surface composition and inductively coupled plasma-mass spectrometry (ICP-MS) was used to analyze bulk composition of the catalysts and to demonstrate the insertion of alkali metal halides in the PHI framework. The spectra confirm the presence of C, N, O, K, Cl, and Li elements in MX → PHI while only C, N, and O were present in BCN, clearly indicating the doping of MX into MX → PHI (Figure S11). The high resolution C 1s spectrum in MX → PHI showed three peaks at 288.3, 286.4, and 284.9 eV (Figure 2a C 1s). The peaks at 288.3 and 284.9 eV are attributed to C atoms in aromatic N–C=N structures and graphitic C–C, respectively, and are present in both MX → PHI and BCN (Figure S12). However, the peak at 286.4 eV (Figure 2a C 1s) in MX → PHI originates from the C≡N species, as was later corroborated by FTIR analysis. The N 1s XPS spectra (Figure 2a N 1s) display 4 peaks: the peaks at 398.5 and 399.9 eV are assigned to the N atoms within C–N=C and N–(C)_3_ in heptazine units; the peak at 401.1 eV belongs to the N atoms in C≡N species or bridging −NH_*x*_. Thus, XPS results also confirmed the existence of heptazine frameworks in MX → PHI. Two peaks (293.0 and 295.8 eV) with a doublet separation value of 2.8 eV of K 2p showed the presence of K in MX → PHI framework. The XPS spectra of Cl 2p and Li 1s were also measured and the peak assignments confirmed the presence of Cl and Li (Figure 2a). Overall, these results from XPS, STEM-XEDS, and ICP-MS (Table S1) showed the uniform and successful insertion of MX into the PHI framework.

The crystal structure of MX → PHI was characterized by powder XRD measurements. A comparison of XRD patterns of MX → PHI_350_, MX → PHI_500_, and MX → PHI (Figure S13a) showed the appearance of additional diffraction peaks and peak shifts in MX → PHI, while some peaks, initially observed in MX → PHI_350_, disappeared with increased temperature. The high-intensity diffraction peaks at 8.3° (10.57 Å) and 27.5° (3.25 Å) in the XRD pattern of MX → PHI confirmed the poly(heptazine imide) structure of MX → PHI (Figure 2b–d). The XRD patterns demonstrated that, relative to the 3.20 Å interplanar stacking in the triazine structured BCN (Figure S13b), there is a slightly wider interplanar stacking (3.25 Å) of poly(heptazine imide) units in the perpendicular direction and heptazine unit stacking with about 10.57 Å in-plane periodicity, which are driven by the insertion of MX. The results show that the triazine phase is further polymerized into the polyheptazine phase through a controlled ionothermal polymerization process in the presence of MX under an Ar atmosphere. Thus, the potential changes in interplanar stacking, together with the possible adjustments in the electron-rich π conjugated framework, the in-plane lattice packing, and the edge defects resulting in −C≡N and −NO_*x*_ functionalization are affirmed upon intercalation of alkali-metals and halide ions.^40−42^

**Figure 3:**
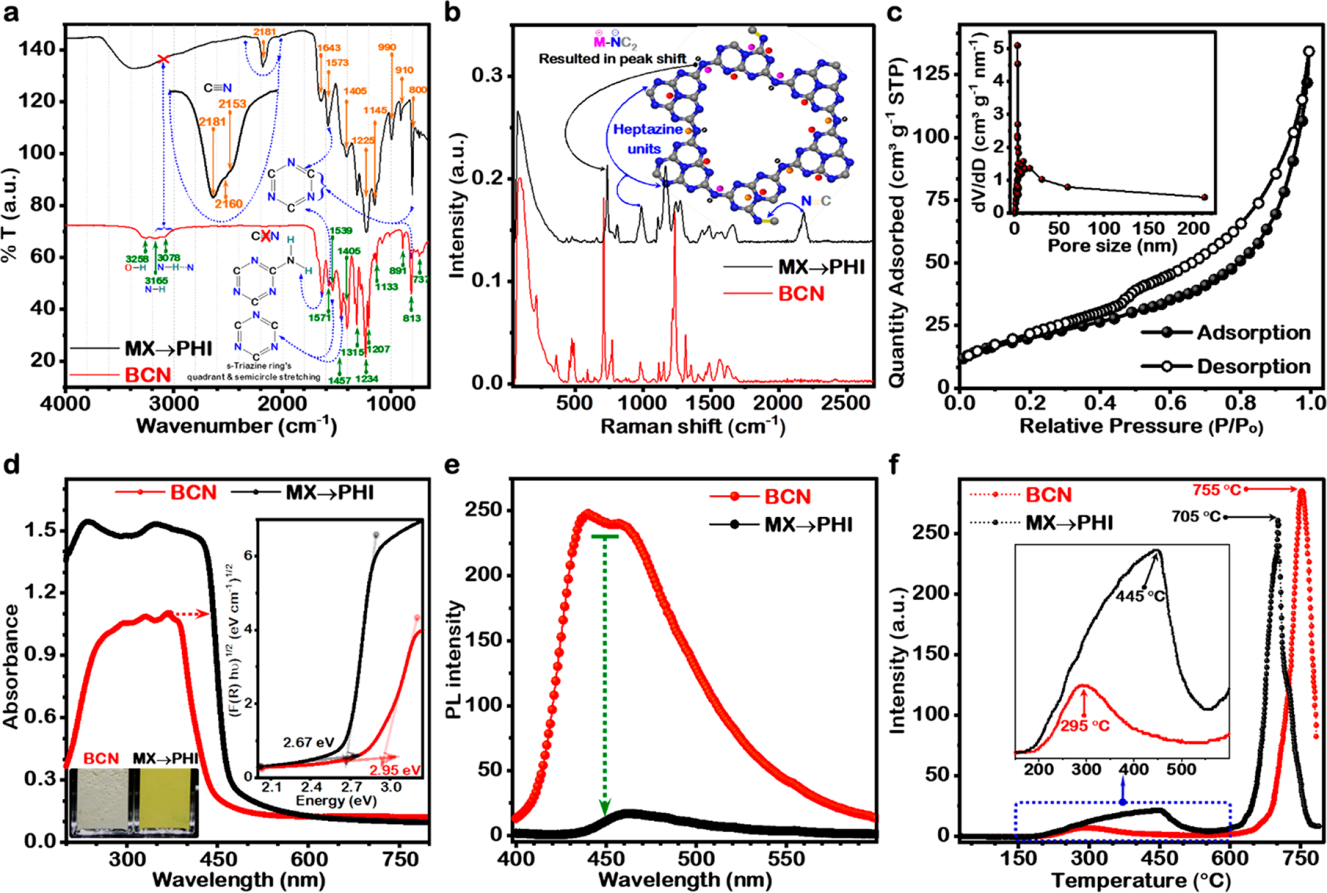
Functionalized, enhanced light absorption, band gap modulated, and O_2_ confined MX → PHI porous material. (a) ATR-FTIR and (b) Raman spectra highlight the distinct functionality (−C≡N) and charge coordination over the MX → PHI framework in contrast to BCN. (c) N_2_ adsorption–desorption isotherm and Barrett–Joyner–Halenda (BJH) pore size distribution (inset) plot of MX → PHI. (d) UV–vis absorbance spectra for BCN and MX → PHI highlighting the increase in light absorbance and a red shift in the absorbance spectrum (red dotted arrow pointing right) for the latter. Inset optical images highlight the color variation. Kubelka–Munk plots (inset) for the bandgap calculations. (e) Photoluminescence spectra for BCN and MX → PHI. (f) O_2_ TPD profiles of BCN and MX → PHI.

Attenuated total reflectance coupled Fourier transform infrared (ATR-FTIR) (Figure 3a and Figure S14), and Raman (Figure 3b and Figure S15) spectroscopic techniques were used for the characterization of BCN, MX → PHI_350_, MX → PHI_500_, and MX → PHI, so that the thermal transformation of urea to MX → PHI in the presence of alkali metals halides could be confirmed (as discussed in Supporting Note S1).

To explore further the textural properties and to provide confirmation of the porous geometry of the 3D hollow fibers/rods in MX → PHI particles, as sketched in Figure 1, N_2_ adsorption–desorption measurements were performed at 77 K and isotherms have been reported and discussed in Figure 3c and Figure S16 and Supporting Note S2. The results of SEM, TEM, XPS, XRD, FTIR, and N_2_ adsorption–desorption together demonstrate the successful preparation of an alkali metal halide incorporated poly(heptazine imide) photocatalyst. To test further the suitability of MX → PHI as an efficient photocatalyst for increased photocatalytic H_2_O_2_ production, the optical properties and charge separation ability of the material were analyzed.

### 2.2 Optical Properties and Electronic Band Structure

The high light absorption efficiency of MX → PHI was confirmed by diffuse reflectance UV–visible (DR-UV vis) absorption spectroscopy, reported in Figure 3d. Compared with BCN, the MX → PHI hollow fibers/rods show significantly higher light absorption, both in the UV and visible regions (Figure 3d) as well as a red-shift (Figure 3d, highlighted by the red arrow). The red-shift in the absorption spectrum of the MX → PHI photocatalyst suggests extended π conjugation, and a delocalized aromatic π conjugated system.^43,44^ The high light absorption efficiency of MX → PHI is probably due to multiple diffuse reflectance inside the nanoarchitecture, leading to trapping and deep penetration of solar radiation. (Figure 1 and Figure S5).

The inset digital images (Figure 3d) of BCN and MX → PHI samples show an apparent color change from white to greenish yellow, which suggests that the bandgap is altered in MX → PHI resulting in extended solar spectrum absorption efficacy. The UV–vis absorption spectra also highlighted an intense band between 350 and 450 nm assigned to a π → π* transition in the s-triazine unit of the C–N based polymers.^38,45^ The visible region absorption edge and steep UV–vis absorption spectrum for MX → PHI (Figure 3d) demonstrate the high purity of the light absorber and the UV–vis absorption results from the band gap transition. These transitions are attributed mainly to charge transfer from the filled valence band (VB) of the N 2p orbital to the conduction band (CB) of the C 2p orbital. Furthermore, the band gap energy (*E*_BG_) calculated using the Kubelka–Munk function: (*F*(*R*)*hν*)^1/2^ = *hν* (Figure 3d, inset) for MX → PHI (2.67 eV) is lower than that for BCN (2.98 eV), which is consistent with their band edge wavelengths and demonstrates that the MX → PHI is viable as a visible light adsorber photocatalyst. These results indicate that structure modulation by alkali metal halides can reduce the band gap and increase the light harvesting ability of MX → PHI.

In addition to bandgap engineering, the band edge positions (VB and CB) also have great significance for the efficient use of photogenerated charge carriers to perform specific redox reactions. The estimated valence band energies (*E*_VB_) for BCN and MX → PHI photocatalysts, measured by UPS analysis, are 6.72, and 7.02 eV, respectively (Figure S17).^46^ After determining the *E*_VB_, the conduction band energy (*E*_CB_) for respective photocatalysts is estimated by the relation *E*_CB_ = *E*_VB_ – *E*_BG_; and the *E*_VB_, *E*_CB_, and *E*_BG_ values are schematically illustrated in Figure S18. This band structure is evidence of a positive shift in the band positions for MX → PHI relative to BCN, with better-aligned energy levels for PCORR to produce H_2_O_2_ and water oxidation or organic molecule oxidation (Figure S18). In particular, the more positive CB value possibly enhanced the photochemical O_2_ reduction capability of MX → PHI to generate significant amounts of H_2_O_2_.

Room-temperature PL emission spectra were recorded under excitation at 350 nm for BCN and MX → PHI (Figure 3e) to probe the separation and recombination of photogenerated charge carriers. As shown in Figure 3e, BCN exhibited an intense emission peak centered around 450 nm, which highlighted the higher recombination rate of the photogenerated charge carriers. In contrast, a marked drop in the peak intensity and a flat emission spectrum were observed for MX → PHI (Figure 3e), which indicate a suppressed electron–hole pair recombination rate and enhanced charge carrier separation efficiency. Thus, the MX → PHI photocatalyst clearly inhibits the different radiative charge carriers’ recombination pathways, associated with aromatic structured photocatalysts.

The accumulation of O_2_ gas molecules around the active sites on the photocatalyst surface can facilitate the 2e^–^ pathway of PCORR to H_2_O_2_ (O_2_ + e^–^ → O_2_^•–^, with superoxide anion radicals as a reaction intermediate; O_2_^•–^ + 2H^+^ + e^–^ → H_2_O_2_), and is possibly one of the primary causes of the exceptional photochemical performance of MX → PHI. Therefore, preliminary thermal studies were conducted to gain information about the interaction or encapsulation of O_2_ into the porous structured, polymerized heptazine units, and the interplanar stacking of the ion MX → PHI photocatalyst. In addition, we may compare with the BCN samples to highlight the superior photoactivity of MX → PHI toward PCORR. The temperature-programmed deoxygenation (O_2_ TPD) and TGA profiles for both the materials are reported in Figure 3f and Figure S19, respectively. The O_2_ TPD profiles (Figure 3f) for both the materials exhibit two distinctive peaks in the temperature range of 150–550 °C and 650–800 °C. The release of chemisorbed and surface lattice O_2_ molecules resulted in deoxidation peaks with maxima at 295 and 445 °C for BCN and MX → PHI (Figure 3f, inset), respectively. As well as a significant peak shift to higher temperature for MX → PHI photocatalyst, the amount of desorbed O_2_, based on the peak area, is also ∼4 times higher than for the reference BCN photocatalyst. The positively charged alkali metal encapsulated into the C–N based PHI framework can play a key role in the interaction of O_2_ with the surface of the molecular photocatalyst. The TGA thermograms (Figure S19) further complemented the O_2_ TPD results, as a steady weight loss in the temperature range of 25–550 °C was observed for MX → PHI, possibly because of adsorbed water molecules and atmospheric gases.

Since light harvesting, energy band structure, surface area, and charge carrier separation efficiency are the main factors affecting the performance of photocatalysts, from the factors discussed above, we would expect a significantly enhanced solar H_2_O_2_ production for MX → PHI. The mesoporous character, higher surface area, lamellar hollow fiber structure of MX → PHI, and the presence of alkali metal halides contributed to improving the light absorbance efficiency, the charge carrier separation, and the O_2_ gas molecule confinement, which in turn should improve the photochemical performance of MX → PHI for the O_2_ reduction reaction.

### 2.3 Solar H_2_O_2_ Production

**Figure 4:**
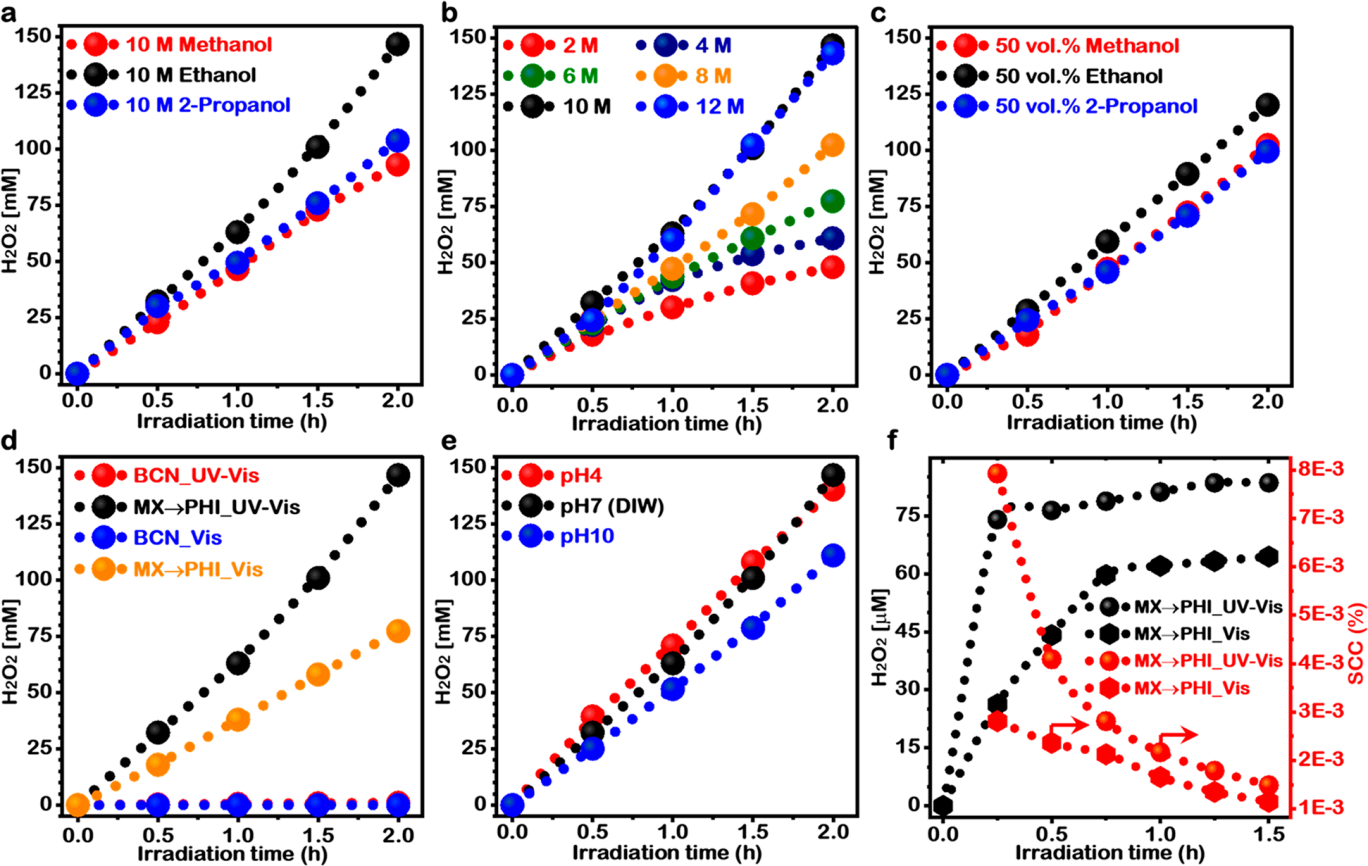
Photochemical H_2_O_2_ production performance of MX → PHI. (a) Plot of H_2_O_2_ production over time using different reaction solvents (C_1_–C_3_ aliphatic alcohol). (b) H_2_O_2_ production over time for different molar concentrations of ethanol. (c) Typical time course H_2_O_2_ production in three different solvents at fixed water content (50 vol %). (d) Comparison of O_2_ reductive solar H_2_O_2_ production of BCN and MX → PHI under UV–visible and visible (≥400 nm) light irradiation. (e) Effect of solution pH on solar H_2_O_2_ production. (f) Time profile solar H_2_O_2_ production and solar-to-chemical conversion (SCC) efficiency from H_2_O and O_2_ under UV–visible and visible light irradiation.

The photochemical H_2_O_2_ production performance of the visible light absorber MX → PHI (*E*_BG_ = 2.67 eV) has been comprehensively investigated under UV–visible light. First, to optimize the reaction conditions of the particulate photochemical system for maximized solar H_2_O_2_ production on MX → PHI, a variation in reaction solvent was examined. The solar H_2_O_2_ production profiles for three different electron and proton donor aliphatic alcohols at a fixed concentration of 10 M are reported in Figure 4a, demonstrating that ethanol is an optimal solvent for solar H_2_O_2_ production. The highest solar H_2_O_2_ production of 146.8 mM was achieved for 2 h irradiation of MX → PHI in 10 M ethanol.

Furthermore, six different concentrations of ethanol in solution were analyzed for maximized solar H_2_O_2_ production (Figure 4b). As expected, a steady increase in solar H_2_O_2_ production rate was observed with an increase in the concentration of ethanol. After 2 h of the photochemical reaction, the recorded solar H_2_O_2_ production rate in 2 M ethanol solution was 24.0 mM h^–1^, which further increased to 30.4 mM h^–1^ for 4 M, 38.7 mM h^–1^ for 6 M, 51.2 mM h^–1^ for 8 M, and finally reached a maximum of 73.4 mM h^–1^ for the 10 M ethanol solution. For higher concentrations, no significant increases in the solar H_2_O_2_ production rate was observed and a nearly constant value of 71.46 mM h^–1^ for 12 M ethanol was recorded. These results show that a proper balance can be achieved between the generated ions (H^+^ and O_2_^•–^) and their mobilities in the liquid phase to perform the proton and electron transfer for the solar H_2_O_2_ production reaction (O_2_^•–^ + 2H^+^ + e^–^ → H_2_O_2_), using a 10 M ethanol solution. An excess of electron and proton donors may impede the surface reaction at the solid–liquid interface, preventing the accessibility of reactants and the mobility of charged reaction intermediates. An additional experiment with different aliphatic alcohols having a fixed water content (50 vol %) was also performed (Figure 4c and Supporting Note S3). Furthermore, to identify the optimized photocatalyst concentration in the reaction solution, three different suspension concentrations of MX → PHI (0.5, 1.0, and 1.5 g L^–1^) in 10 M ethanol were investigated and reported in Figure S20.

The time-dependent solar H_2_O_2_ production profiles for triazine-based BCN and heptazine imide-based MX → PHI in 10 M ethanol solution (Figure 4d) provide a comparison of their PCORR capabilities. Clearly, triazine-structured BCN displayed a poor efficiency toward 2e^–^ PCORR pathway for solar H_2_O_2_ production and only generated 0.98 mM H_2_O_2_ after 2 h of UV–vis irradiation (Figure S21). In contrast, the alkali metal halide-structure modulated, surface-functionalized MX → PHI demonstrated a significantly higher solar H_2_O_2_ production (146.8 mM) at a rate of 73.4 mM h^–1^. The latter is the highest reported value for solar H_2_O_2_ production that we have found among other particulate photochemical systems, irrespective of photocatalyst type (carbon nitride, metal oxide, metal sulfide, metal organic-based, hybrid, etc.).^11,47^ Additionally, the effectiveness of MX → PHI under the visible light spectrum was also analyzed. For this, solar H_2_O_2_ production was carried out in O_2_ saturated 10 M ethanol solution under visible light irradiation (≥400 nm), while keeping all other conditions the same. Even under visible light irradiation (Figure 4d), MX → PHI gave a high yield of solar H_2_O_2_ (77.3 mM), which is also the highest among those reported using particulate photochemical systems, so far.^11,47^

Thus, MX → PHI resulted in nearly 150 times higher solar H_2_O_2_ production than that of BCN under UV–visible irradiation and >4250 times higher solar H_2_O_2_ production as compared to BCN under visible light irradiation. As discussed earlier (Figure 1), the significantly enhanced photocatalytic performance of MX → PHI is a consequence of a combination of factors: the synergistic effect of the morphology and optical and electronic properties induced by the structure-modulation of poly(heptazine imide) with alkali metal halides through controlled ionothermal polymerization.

The pH of the reaction solution may also have a significant effect on the proton-coupled electron transfer-assisted solar H_2_O_2_ production. Therefore, the photocatalytic production of solar H_2_O_2_ was also carried out at pH 4 and pH 10 (Figure 4e). The MX → PHI showed a significant decrease in solar H_2_O_2_ production with increased pH (pH 10) whereas an insignificant difference was observed in the H_2_O_2_ production profile for PCORR carried out at pH 4 and neutral pH solution (without maintaining the pH using acid or base). The results show that no additional pH adjustment steps are required to maximize the performance.

The photocatalytic performance of the as-synthesized MX → PHI for reductive solar H_2_O_2_ generation from O_2_ saturated deionized water (DIW) without using any electron and proton donor sacrificial agent was also evaluated to corroborate the greater possibilities and high potential of MX → PHI for unassisted solar fuel production. A significant amount of solar H_2_O_2_ production (74.0 μM) in the initial 15 min of light irradiation over bare MX → PHI was observed under UV–visible light irradiation (Figure 4f), which is also comparable to some of the most recently reported photocatalytic systems.^15,18,32^ A relatively low solar H_2_O_2_ production and SCC efficiency in the absence of a sacrificial agent is probably due to the consecutive decomposition of photogenerated H_2_O_2_ on the MX → PHI surfaces during the photochemical reaction, which explains why the self-oxidation of photogenerated H_2_O_2_ resulted in the saturation of the H_2_O_2_ production after 30 min of irradiation (Figure 4f).

**Figure 5:**
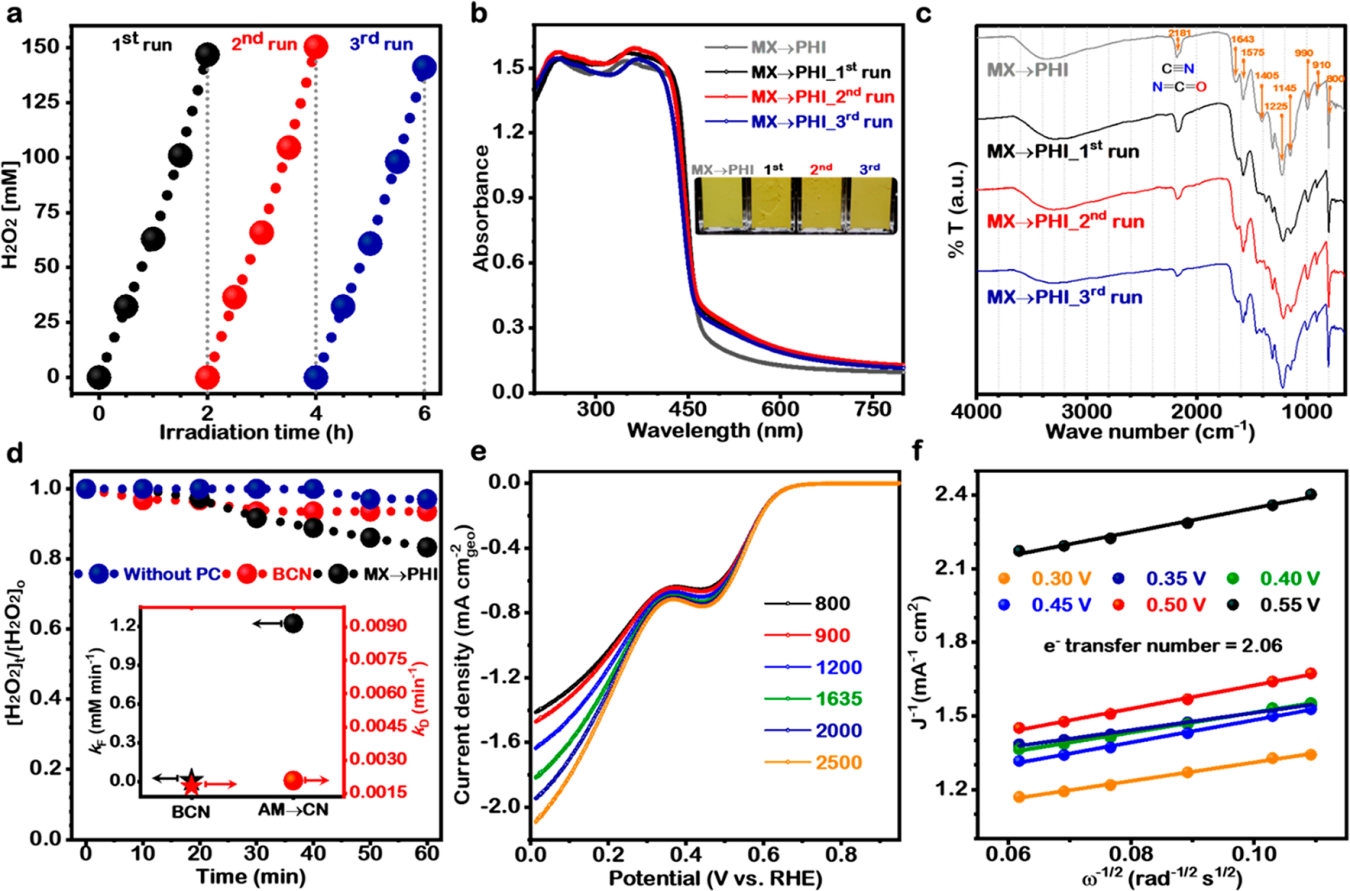
Stability, kinetics, and comparison. (a) Repeated run stability analysis of MX → PHI to highlight the recyclability. (b) UV–vis absorbance spectra of initial MX → PHI, samples recovered from the reaction solution after each cyclic run to confirm the structural stability, and persistent optical properties. The inset optical image of powder samples highlights the sustained color of the photocatalyst. (c) FTIR spectra. (d) Photochemical H_2_O_2_ decomposition over BCN and MX → PHI samples under UV–visible light irradiation. Inset graph highlights the photochemical H_2_O_2_ formation (*k*_F_) and decomposition (*k*_D_) kinetics constant over BCN and MX → PHI. (e) Linear sweep voltammetry plots of MX → PHI in O_2_ saturated 0.1 M KOH at different rotating speeds ranging from 800 to 2500 rpm. (f) K–L plots at different potentials, and the evaluated number of electrons participating in the O_2_ reduction reaction.

The cyclic photocatalytic performance of MX → PHI was examined under the same reaction conditions for three repeated runs (Figure 5a). The linear increase in solar H_2_O_2_ production for each run demonstrated a sustained photoactivity of MX → PHI. To substantiate further the unchanged surface structure and intact optical properties of MX → PHI during the PCORR recyclability tests, the samples collected after each run were analyzed by DR-UV–vis (Figure 5b) and FTIR (Figure 5c) spectroscopy. The FTIR spectra for each collected sample at the end of PCORR did not show any significant change in the vibration peak positioning and their intensities (Figure 5c). However, relative to the original MX → PHI sample, the absorption spectra for MX → PHI collected after the first and second runs displayed a slight improvement in light absorption, with a red-shift and extended tailing (Figure 5b). The extended tail may correspond to minor changes in the surface functionality of the polymeric structure of MX → PHI, as a result of photoactivation during PCORR. The cyclic photochemical performance (Figure 5a), spectroscopic examinations (Figure 5b,c) and N_2_ adsorption–desorption studies (Figure S22) showed that the photoactivity, chemical structure, and texture properties of the MX → PHI photocatalyst remained largely unchanged during the repeated experiments.

The apparent quantum yield (AQY) of MX → PHI for solar H_2_O_2_ production was also measured using 365 and 450 nm light irradiation. The AQY values obtained for H_2_O_2_ production at 365 nm (UV light) and 450 nm (visible light) are ∼96% and 21%, respectively, for MX → PHI with a catalyst dosage of 1 g L^–1^ in 10 M ethanol (Figure S23). These values are higher than those reported for previous photocatalysts for peroxide production, indicating that MX → PHI is a highly efficient molecular photocatalyst for sustainable solar H_2_O_2_ production via a 2e^–^ pathway. AQY values for PCORR to H_2_O_2_ matched well with the DR-UV visible spectrum of MX → PHI proving that the PCORR is via a 2e^–^ process.

The reaction kinetics of 2e^–^ PCORR to H_2_O_2_ over the surface of irradiated BCN and MX → PHI photocatalysts was investigated using the kinetic model of photochemical H_2_O_2_ generation at the initial phase of reaction reported by Hoffmann and co-workers as follows: [H_2_O_2_] = (*k*_F_/*k*_D_)(1 – e^–*k*_D_*t*^) + [H_2_O_2_]_0_ e^–*k*_D_*t*^.^48,49^ Here, *k*_F_ and *k*_D_ are the rate constants for photochemical H_2_O_2_ formation and decomposition reactions, respectively. Following the reaction kinetics, the H_2_O_2_ formation rate is determined by zero-order kinetics because the reaction solution is continuously purged with O_2_, while the decomposition reaction rate with fixed initial H_2_O_2_ concentration follows first order kinetics. The *k*_D_ value for MX → PHI (0.00208 min^–1^) (Figure 5d, inset), obtained after fitting the H_2_O_2_ photodecomposition profile (Figure 5d) to first-order reaction kinetics, was slightly greater than that of triazine structured BCN (0.00183 min^–1^). However, a large difference between *k*_F_ values of MX → PHI (1.2233 mM min^–1^) and BCN (0.0085 mM min^–1^) was observed. The kinetic data demonstrate that solar H_2_O_2_ production is primarily governed by the formation kinetics. Furthermore, electrochemical rotating disc electrode (RDE) analysis confirms the 2e^–^ O_2_ reduction pathway to H_2_O_2_ generation rather than 4e^–^ (H_2_O formation) over MX → PHI (Figure 5e,f). The calculated electron transfer number from the slopes of the linearly fitted Koutecky–Levich (K–L) plots at the different potentials (Figure 5f) was around 2.06.

Furthermore, to validate the generation of O_2_^•–^ (superoxide anion radical) intermediate reaction species during photochemical H_2_O_2_ production over the MX → PHI surface, the *in situ* coloration of XTT (2,3-bis(2-methoxy-4-nitro-5-sulfophenyl)-2H-tetrazolium-5-carboxanilide) when reacted with photogenerated O_2_^•–^ into orange colored XTT-formazan (Figure S24a) has been used. The appearance of the dark orange color (Figure S24b) and absorbance λ_max_ around 481 nm (Figure S24c) illustrated the generation of the O_2_^•–^ reaction intermediate during PCORR over MX → PHI.

Considering the exceptional solar H_2_O_2_ production performance of MX → PHI via the 2e^–^ PCORR pathway in an organic solvent, a comparison was drawn with previously reported photocatalysts for similar reaction systems. The present MX → PHI photocatalyst exhibited a higher solar H_2_O_2_ production rate than that of most of the carbon nitride, metal oxide, metal sulfide, and metal organic-based photocatalysts, respectively (Table S2).

## 3 Summary and Conclusions

We successfully achieved the highest ever solar H_2_O_2_ production rate (73.4 mM h^–1^) via the 2e^–^ PCORR pathway on an alkali metal-halide modulated poly(heptazine imide) (MX → PHI). Compared to the triazine structured pristine carbon nitride, there is an increase of nearly 150 and >4250 times in H_2_O_2_ production on MX → PHI under UV–visible and visible light (≥400 nm) irradiation, respectively, which reflects the effect of the basic structure of poly(heptazine imide) and the engineering of its morphological, optical, and electronic properties via alkali metal-halides. In particular, combining effective light absorption, charge separation, and O_2_ trapping in MX → PHI makes it an exceptionally highly photoactive molecular catalyst. Our study provides insight for potential materials based on poly(heptazine imide) for sustainable H_2_O_2_ production by utilizing natural resources (sun, water, and air).

## 4 Experimental Section

### 4.1 Synthesis of Bulk Triazine Structured Carbon Nitride (BCN)

The BCN was synthesized via thermal pyrolysis of urea at 550 °C for 3 h in a muffle furnace. After the completion of thermal polymerization of urea to triazine structured g-C_3_N_4_, the product was washed with deionized water and collected by filtration followed by vacuum drying. The dried white product (4.6% yield with respect to urea precursor) was further ground to a fine powder and stored as such for photochemical performance evaluation.

### 4.2 Synthesis of Alkali Metal-Halides (MX) Modulated PHI (MX → PHI)

The MX → PHI was synthesized by controlled ionothermal polymerization processes. The distinctly structured MX → PHI was obtained by mixing a fixed ratio of urea to KCl–LiCl eutectic mixture (5:3) to carry out the polymerization in a tube furnace under a continuous flow of Ar gas at 550 °C at a ramp rate of 3 °C min^–1^ for 5 h. The synthesis is sensitive to atmospheric conditions, therefore, Ar gas was continuously purged into the reaction mixture to minimize the O_2_ and water content. The greenish-yellow colored product, obtained from the cooled polymerized sample, was washed with DI water and collected by filtration followed by vacuum drying. The dried product was ground into a fine powder with an agate mortar and stored as such in an amber vial for photochemical studies and characterization. The final yield of MX → PHI (7.5% with respect to urea precursor) was higher than BCN. Ionothermal polymerization facilitates more uniform doping in the basic framework of the PHI molecular photocatalyst and simultaneously might introduce surface functionality and performance-enhancing structural defects. Moreover, ordering and stabilization of the intermediates result in the synthesis of a highly efficient MX → PHI molecular photocatalyst.

### 4.3 Photochemical H_2_O_2_ Production

For particulate photochemical experiments, a fixed concentration of 7.5 mL of alcohol solution (C_1_–C_3_) was placed in the Pyrex glass test tube, followed by the addition of 7.5 mg of photocatalyst (except an experiment including photocatalyst concentration variation). The reaction suspension was subjected to light irradiation with continuous O_2_ gas bubbling (∼100 cc) throughout the experiment for PCORR. The photochemical performance of BCN and MX → PHI molecular photocatalysts were examined and compared under UV–visible as well as visible light only (≥400 nm) using a 150 W xenon lamp (optical irradiance 175 mW cm^–2^) coupled with an air mass filter (AM1.5G). During the photochemical reaction, 0.5–1.0 mL aliquots were collected at certain time intervals by a syringe and the clear sample was obtained by using a 0.20 μM pore, 15 mm Minisart RC, syringe filter. For the cyclic performance of photocatalysts, 50 mg of photocatalyst suspension (1 g L^–1^) in 10 M ethanol solution was used so that after each run enough material could be collected. After 2 h of irradiation, photocatalysts were filtered out from the reaction mixture, washed with DIW, vacuum-dried, and redispersed in 10 M ethanol solution by keeping the same catalyst concentration (1 g L^–1^).

### 4.4 Hydrogen Peroxide Detection

The H_2_O_2_ concentration in the aliquot collected at different time intervals from the reaction suspension during light irradiation was measured by the DPD colorimetric method using a UV–visible spectrophotometer (UV-1800, Shimadzu).^18^ Depending on the H_2_O_2_ concentration, the collected samples were diluted multiple times (10–6000) before its estimation so that the photogenerated H_2_O_2_ concentration lies in the calibrated range. To perform the colorimetric estimation of H_2_O_2_ in an aqueous solution, 0.4 mL of 0.1 M sodium phosphate buffer (pH 6) was mixed with 1.12 mL of DIW followed by the addition of 1 mL of sample. To the buffered solution, 0.05 mL of *N*,*N*,-Diethyl-*p*-phenylene-diamine sulfate (DPD) solution followed by 0.05 mL of peroxidase (POD) was mixed to catalyze the oxidation of DPD in the presence of H_2_O_2_ to generate a pink color due to radical cations as shown in Figure S25. The resultant colored solution was used for spectrophotometric measurement of H_2_O_2_ concentration at λ_max_ 551 nm using an external standard curve (*R*^2^ > 0.998). Moreover, a zero/blank reading for reaction suspension was conducted with the aliquot collected before irradiation for accurate quantification of photogenerated H_2_O_2_ in each experiment.

The apparent quantum yield (AQY) for solar H_2_O_2_ production was calculated using the following equation:
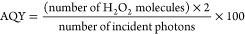


The solar-to-chemical conversion (SCC) efficiency^50^ for H_2_O_2_ production from H_2_O and O_2_ (2H_2_O + O_2_ + *h*ν → 2H_2_O_2_: Δ*G*° = 117 kJ mol^–1^) (i.e., in the absence of a sacrificial agent) was determined using the same reaction setup used for other particulate photochemical experiments as discussed in previous sections. In a typical photochemical 7.5 mg of MX → PHI photocatalyst was dispersed in 7.5 mL of DIW. The resultant reaction suspension in the Pyrex glass test tube was subjected to side-light irradiation with continuous O_2_ gas bubbling (∼100 cc) throughout the PCORR.
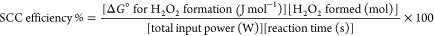


The optical irradiance was 175 mW cm^–2^ and the irradiated area was 5.2 cm^2^. During the PCORR, the clear liquid samples were collected at fixed time intervals by using a 1 mL of syringe followed by syringe filtration (0.20 μM pore, 15 mm Minisart RC, syringe filter). The H_2_O_2_ amount in the solution was quantified by DPD colorimetric method using a UV–visible spectrophotometer.

### 4.5 Electrochemical Analysis

To study the ORR kinetics, Koutecky–Levich plots (J^–1^ vs ω^–1/2^) were derived from linear sweep voltammetry (LSV) at room temperature using a rotating disk electrode (RDE) setup from Metrohm connected to an Autolab potentiostat. The cell consists of an Ag/AgCl electrode in saturated KCl (3 M) aqueous solution as the reference electrode, a Pt sheet as the counter electrode, and glassy carbon (GC) electrode with a geometric area of 0.196 cm^2^ as the working electrode. The electrochemical measurements of the catalysts were performed in 0.1 M KOH electrolyte under continuous O_2_ purging. To prepare a homogeneous catalyst ink, 10 mg of photocatalyst and 68.7 μL of Nafion solution were dispersed in 600 μL of 2-propanol by sonication for 30 min. Then, 10 μL of the catalyst ink was then loaded on glassy carbon as the working electrode and dried in an oven at 50 °C. The LSV’s for resultant working electrode were measured in the potential range of 0.0 to 0.95 V_RHE_ at a scan rate of 5 mV s^–1^ and at different rotating speeds (800, 900, 1200, 1625, 2000, and 2500 rpm).

Koutecky–Levich plots (K–L) were analyzed at various electrode potentials. The slopes (*B*^–1^) of their linear fit lines were used to calculate the number of electrons transferred (*n*) based on the Koutecky–Levich equation:
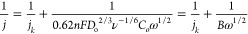


Here *j* indicates current density (mA cm^–2^), *j*_*k*_ kinetic current density (mA cm^–2^), *n* electron transfer number (*n*), *F* Faradaic constant (96485 C mol^–1^), *D*_o_ diffusion coefficient of dissolved oxygen in the 0.1 M KOH at 298 K (1.9 × 10^–5^ cm s^–1^), ν kinematic viscosity of the 0.1 M KOH (0.01 cm^2^ s^–1^), *C*_o_ saturation concentration of dissolved oxygen in the 0.1 M KOH (1.2 × 10^–3^ mol L^–1^), and ω angular velocity of the disk electrode (rad s^–1^). The slope (*B*^–1^) of the plot *j*^–1^ as a function of ω^–1/2^ is used to calculate the electron transfer number (*n*).

## Supporting Information Available

Materials details; characterization information; Supporting Note 1, ATR-FTIR and Raman spectroscopy; Supporting Note S2, N_2_ adsorption–desorption; Supporting Note S3, effect of different aliphatic alcohols having fixed water content (50 vol %) on solar H_2_O_2_ production; additional data on the characterization of as-synthesized samples SEM micrographs, XEDS overlay images, TEM/HRTEM images, FFTs patterns, XPS survey scan/core level spectra, XRD patterns, FTIR spectra, Raman spectra, N_2_ adsorption–desorption isotherm, UPS spectra, energy diagram, and TGA thermograms; photochemical H_2_O_2_ production plots; absorbance spectra and calibration curve for spectrophotometric quantification of photogenerated H_2_O_2_ content in the reaction solution, elemental composition table, and comparison table of photocatalysts for solar H_2_O_2_ production from an oxygen saturated solution of electron and proton donor solvents (PDF)

## Abbreviations

MX → PHI: alkali metal-halide modulated poly(heptazine imide)
BCN: basic carbon nitride
PCORR: photochemical O_2_ reduction reaction
DIW: deionized water.
