# Supplementary material for: Enhanced H2O2 Production via Photocatalytic O2 Reduction over Structurally-Modified Poly(heptazine imide)
Source: Chem Mater. 2022 Jun 8;34(12):5511–21. doi: 10.1021/acs.chemmater.2c00528 (PMC9245186; doi:10.1021/acs.chemmater.2c00528)
Supplement: Supplementary file 1 — cm2c00528_si_001.pdf [file cm2c00528_si_001.pdf]

SUPPORTING INFORMATION (SI)

Enhanced H_2O_2 Production via Photocatalytic O_2 Reduction over Structurally-Modified Poly(heptazine imide)

Pankaj Sharma,*[a,b] Thomas J. A. Slater,[a] Monika Sharma,[c] Michael Bowker,*[a,b] and C. Richard A. Catlow,*[a,b,d]

[a]*Cardiff Catalysis Institute, School of Chemistry, Cardiff University, Cardiff CF10 3AT, United Kingdom*

[b]*UK Catalysis Hub, Research Complex at Harwell, Rutherford Appleton Laboratory, Harwell, OX11 0FA, United Kingdom*

[c]*Department of Chemistry, Kurukshetra University, Kurukshetra 136 119, Haryana, India*

[d]*Department of Chemistry, University College London, 20 Gordon St., London WC1 HOAJ, United Kingdom*

E-mail: sharmap14@cardiff.ac.uk (P.S.); bowkerm@cardiff.ac.uk (M.B.); catlowr@cardiff.ac.uk (C.R.A.C.).

MATERIALS

The chemicals used for photocatalyst synthesis, H_2O_2 production, and spectrophotometric analysis were as follows: urea ($CO(NH_2)_2$, ≥ 98%, Sigma-Aldrich), anhydrous lithium chloride (LiCl, ≥ 99%, Sigma-Aldrich), anhydrous potassium chloride (KCl, ≥99%, Sigma-Aldrich), methanol (99.8%, Sigma-Aldrich), ethanol (≥ 99.5%, Sigma-Aldrich), 2-propanol (99.7%, Sigma-Aldrich), N,N,-Diethyl-p-phenylene-diamine sulfate (DPD, ≥98%, Sigma-Aldrich), peroxidase (POD, horseradish, Sigma), hydrogen peroxide (30%, Sigma-Aldrich), sodium phosphate dibasic heptahydrate (98-102%, Sigma Aldrich), sodium phosphate monobasic (≥98%, Sigma), 2,3-Bis(2-methoxy-4-nitro-5-sulfophenyl)-2H-tetrazolium-5-carboxanilide (XTT, Alfa Aesar), and 0.1 N sulfuric acid standard solution. All chemicals used in the study were of analytical grade and ultrapure deionized water (DIW) (18.2 μS cm^{-1}) obtained from the μPure system was used throughout the experiments.

CHARACTERISATION

The morphology of as-synthesized photocatalysts was observed by a scanning electron microscope (SEM) imaging (JSM-6610LV, JEOL) after sputter coating with gold. To analyse elemental composition, X-ray energy dispersive spectrometry (XEDS) was utilized. Scanning transmission electron microscope (STEM) imaging and XEDS mapping were performed using a JEOL ARM200F (probe aberration-corrected) transmission electron microscope equipped with a two-detector Centurio XEDS system at an acceleration voltage of 200 kV. Quantification of STEM-XEDS data was conducted using the Cliff-Lorimer methods using standard calculated factors. The spectrum of interest was taken from a region over a hole in the carbon film. Errors were calculated assuming a 10% error in Cliff-Lorimer factor, added in quadrature with statistical errors from total integrated counts in each peak. TEM imaging was performed using a JEOL ARM300F (image aberration-corrected) transmission electron

microscope at an acceleration voltage of 300 kV. TEM images were recorded using a Gatan Oneview camera. MX→PHI samples damaged rapidly under high electron flux conditions, therefore all images were taken with electron fluence below 10 $e^{-}Å^{-2}$. HRTEM simulation was performed using the abTEM python package, with a defocus of -100 nm, 1 μm Cs coefficient, 20 frozen phonon configurations and a thickness of 8 atomic layers.[1] N_2 adsorption−desorption isotherms at 77 K were measured to evaluate the textural properties on an automated surface area and pore size analyser (Quadrasorb *evo*). The powder X-ray diffraction (XRD) (X-per PRO, PANalytical, Netherlands) was used to analyse and compare the crystal structure of the synthesized photocatalysts using Cu-Kα (λ = 1.5418 Å) radiation. A fluorescence spectrophotometer (Cary Eclipse Fluorescence Spectrophotometer, Agilent Technologies) was used to record the photoluminescence (PL) spectra at 350 nm excitation wavelength. The UV-visible diffuse reflectance spectra were recorded with a UV-visible spectrophotometer (UV-2600, Shimadzu) over a wavelength range 200 – 800 nm. The optical band-gap energies for BCN and MX→PHI have been estimated from the UV-DRS absorption spectra by using the Kubelka-Munk function: $(F(r)h\nu)^{1/2} = h\nu$; where hν and F(r) are the photon energy, and Kubelka-Munk function, respectively. To evaluate the surface functionality and compare the surface structure of BCN and MX→PHI, the FTIR spectra were recorded on a Thermo Scientific, Nicolet iS10, infrared spectrometer. Raman spectra were recorded in the wavenumber range of 50-3600 cm^{-1} on Bruker FT-Multi Ram equipped with Nd: YAG laser source of excitation (1064 nm) at a laser power of 100 mW. XPS surface analysis was carried on a Thermo Scientific K-Alpha spectrometer using an Al Kα X-ray source and a double-focusing hemispherical analyser. The pass energy used for the core scans and survey were 48 and 192 eV, respectively. Parameters used for the measurements were: spot size of 400 μm, pass energy of 50 eV, the energy step size of 0.1 eV, 10 scans in the vicinity of the orbital binding energy of the elements of interest. The temperature-programmed deoxygenation (TPD)

experiments were performed on automated flow chemisorption and reactivity analyzer (ChemBET Pulsar, Anton Paar) equipped with a mass spectrometer. Following the standard procedure, before performing TPD analysis, the photocatalysts were degassed at 150 °C for 3 h under He atmosphere followed by equilibration with O_2 for 1 h after at 25 ºC. The TPD measurements were carried out in He gas at a heating rate of 10 °C min^{-1} up to 800 °C. The thermal gravimetric analysis (TGA) was carried out at a heating rate of 10 °C min^{-1} under a nitrogen atmosphere (Thermogravimetric TGA 5500, TA Instruments). Inductively coupled plasma-mass spectroscopy (ICP-MS) measurements were carried out to analyse the Li and K content in MX→PHI sample.

Supporting Note S1. ATR-FTIR and Raman spectroscopy

FT-IR spectra, reported in **Figure 3a**, firmly established the individual structure and identity of each photocatalyst. The FT-IR spectrum of BCN displayed characteristic broad bands centred at 3078, 3165, and 3258 cm^{-1} for symmetric and antisymmetric stretch vibrations of N···H hydrogen bonds, N-H (residual or terminal $-NH_2$ group) and O-H (−OH group) bonds, whereas these vibrational bands were almost eliminated in the corresponding FT-IR spectrum of MX→PHI (**Figure 3a**)[2]. The bands around 800, and 1570 cm^{-1} are due to breathing modes of the triazine units and aromatic CN heterocycles, respectively[3,4]. Furthermore, most of the vibrational bands corresponding to the polymeric triazine structure are missing from the MX→PHI spectrum, as highlighted in **Figure 3a**. Many new sharp peaks appear in the FT-IR spectrum of MX→PHI (**Figure 3a**), which have been labelled to make a comparison. The replacement of terminal $–NH_2$ by -OH groups resulting in surface –OH group grafting (–C–OH) on intercalation of K, Li, and Cl elements have been confirmed by the appearance of extra bands at 990, 1145, and 1372 cm^{-1} in MX→PHI (**Figure 3a**). Moreover, these vibrational peaks are indicators of alkali metal cation presence in the PHI structure. In addition, the stretching

peak for the cyano group (C≡N) at 2181 cm^{-1} with shoulder peaks in MX→PHI is an indicator of surface functionalization as a result of some structural defects. The FT-IR spectra further confirm the structural shifts in the PHI framework, which implies a different arrangement of C and N in comparison to nanosheet-structured BCN. Similar to the FTIR spectra (**Figure 3a**), strong Raman peaks ~2183 cm^{-1} (C≡N), peak shifts to higher wavenumbers (such as 700 cm^{-1} (BCN) to 735 cm^{-1} (MX→PHI)),[5] and the decreased densities of the peak for MX→PHI (**Figures 3b, and S15**) confirmed the assignments made about the polymeric structure of MX→PHI based on FTIR spectroscopic results. Thus, the shifted peak at 735 cm^{-1} for MX→PHI is probably because of the ionic interaction of positively charged alkali metals (K^+ and Li^+) with the negatively charged nitrogen of PHI units.[5]

Supporting Note S2: N_2 adsorption-desorption

A type IV isotherm with an H_3 hysteresis loop at a relative pressure of 0.45-1.0 substantiates the mesoporous character of MX→PHI, as demonstrated by the SEM micrographs (**Figure S5**). The pore size distribution curve for MX→PHI (**Figure 3c, inset**) is typical of the meso-macroporous structure with a wide distribution of pores largely originating from the hollow particles. Further, MX→PHI has a larger BET specific surface area (S_{BET}) of 71.2 m^2 g^{-1} owing to the hollow structure, whereas, the aggregated sheet-like BCN particles have a lower S_{BET} (45.6 m^2 g^{-1}). The porous character, which arises from the 3D hollow fibre morphology, might boost the light-harvesting efficiency through multi-reflection and light penetration processes.

Supporting Note S3: Effect of different aliphatic alcohols having fixed water content (50 vol.%) on solar H_2O_2 production

We noted during the experiments optimising the ethanol concentration (**Figure 4b)** that water content plays a crucial role, so an additional experiment with fixed water content (50 vol.%)

was also performed. The solar H_2O_2 production profile as a function of solvent type and time (**Figure 4c)** showed no significant difference in the solar H_2O_2 production rate for methanol (51.2 mM h^{-1}) and 2-propanol (49.8 mM h^{-1}), whereas ethanol showed a slightly higher value (60.2 mM h^{-1}).

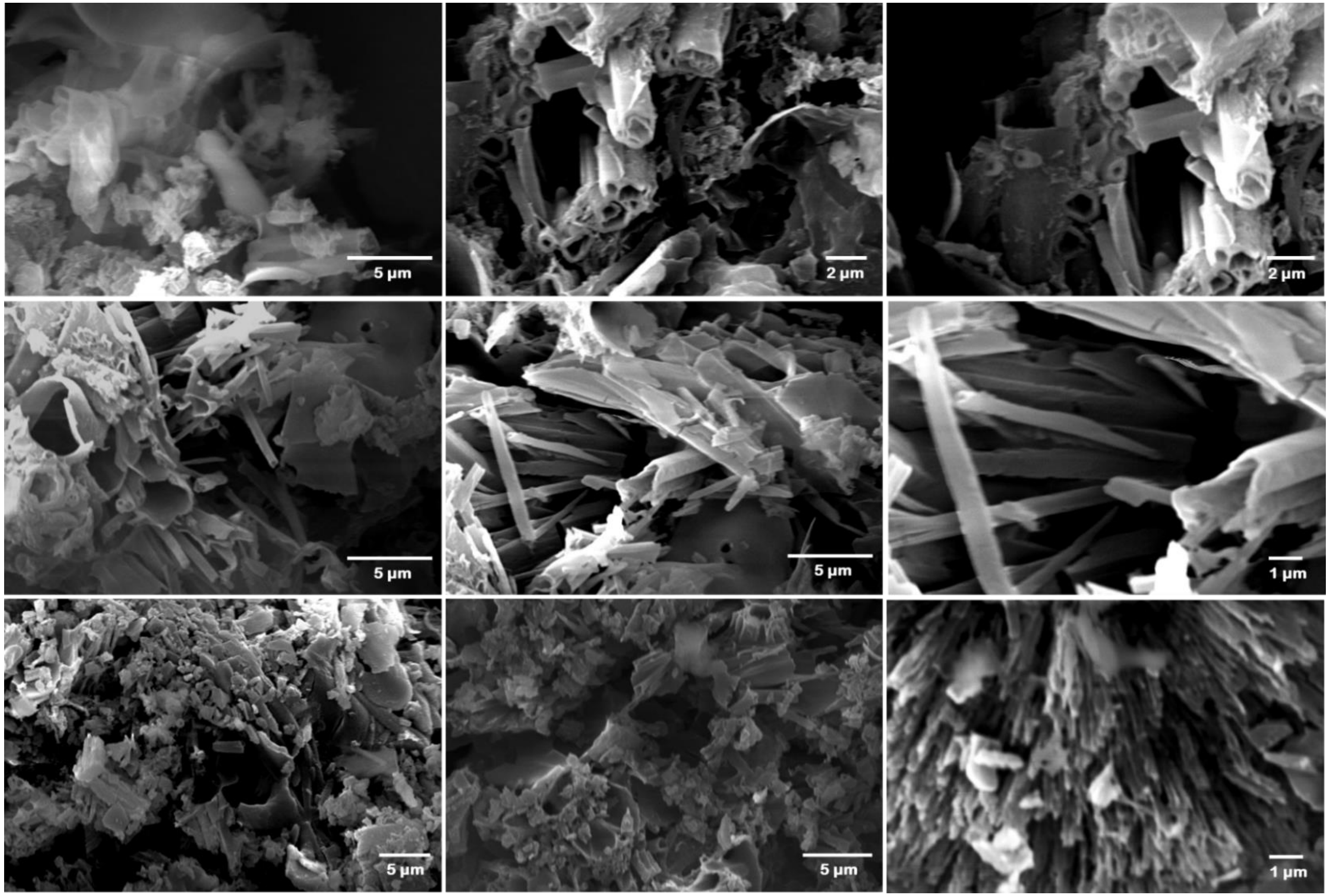

Figure S1. SEM micrographs of MX→PHI_{350} samples highlighting the self-coiling of thin polymeric sheets at the initial stage of MX→PHI polymerisation at 350 °C.

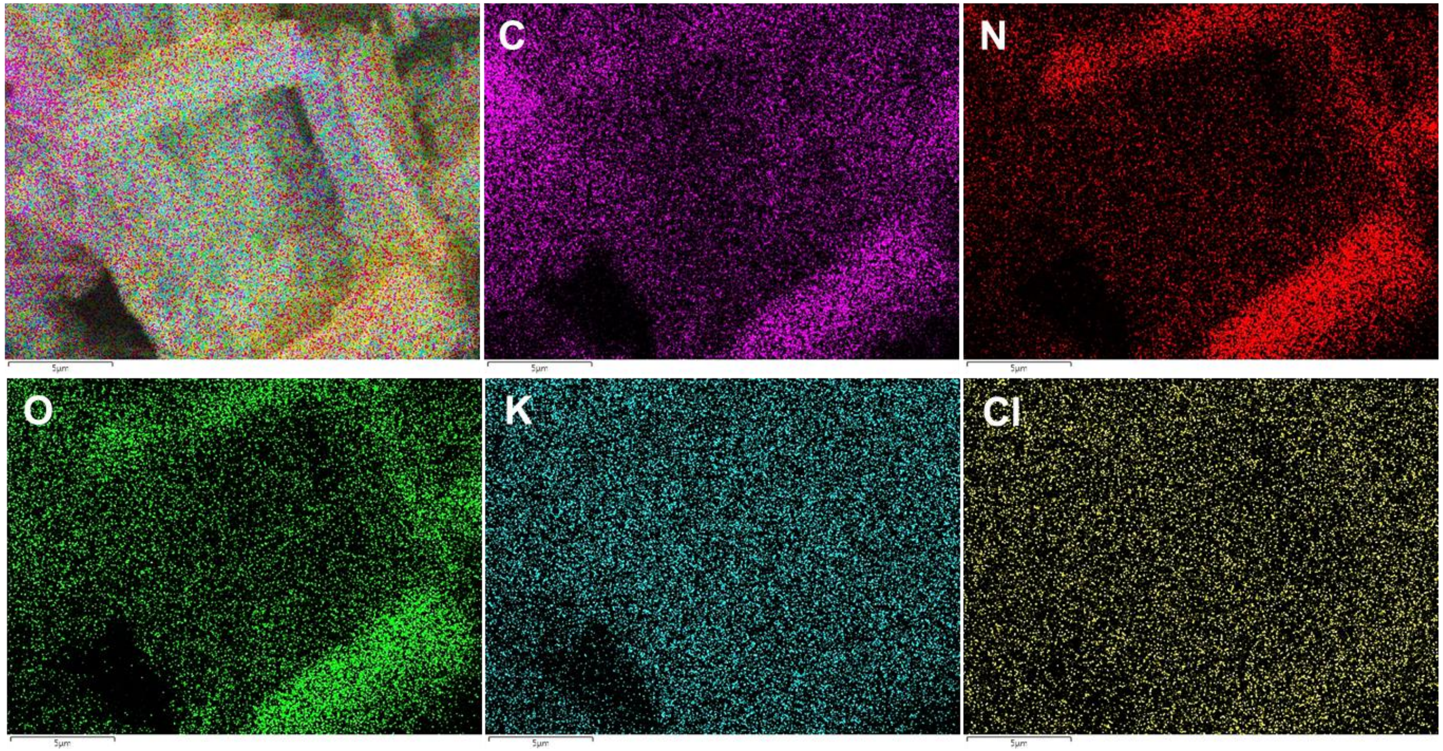

Figure S2. XEDS overlay image and corresponding elemental maps of C, N, O, K, and Cl for a selected area of MX→PHI_{350} sample highlighting the uniform distribution of each element across the sample.

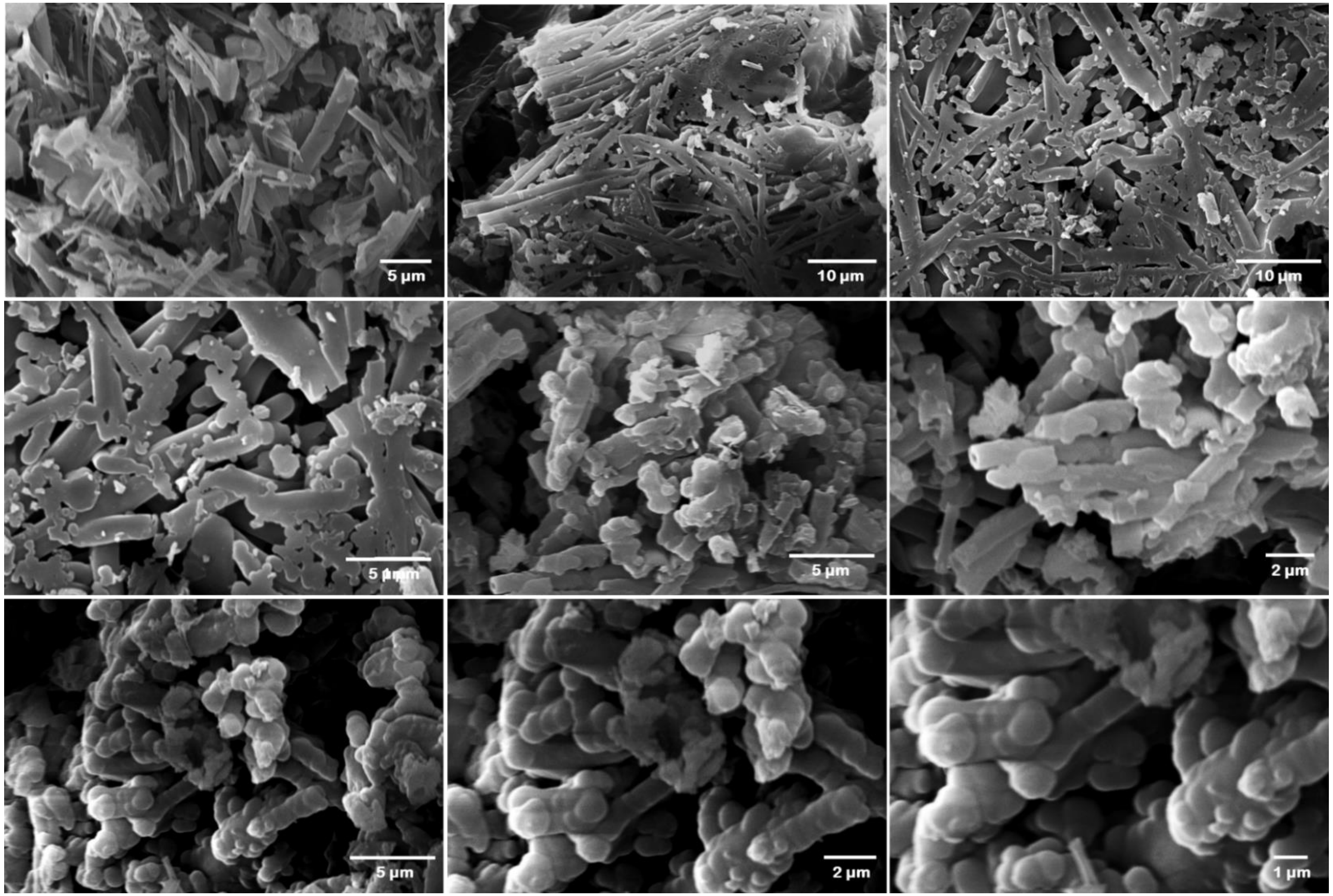

Figure S3. SEM micrographs taken at different magnifications and areas of MX→PHI_{500} sample reveal the growth process of MX→PHI photocatalyst. These micrographs demonstrate that the majority of the samples have thin layered hollow fibre morphology, whereas some of the micrographs highlight the transformation of these hollow fibres/rods to nano-spheres.

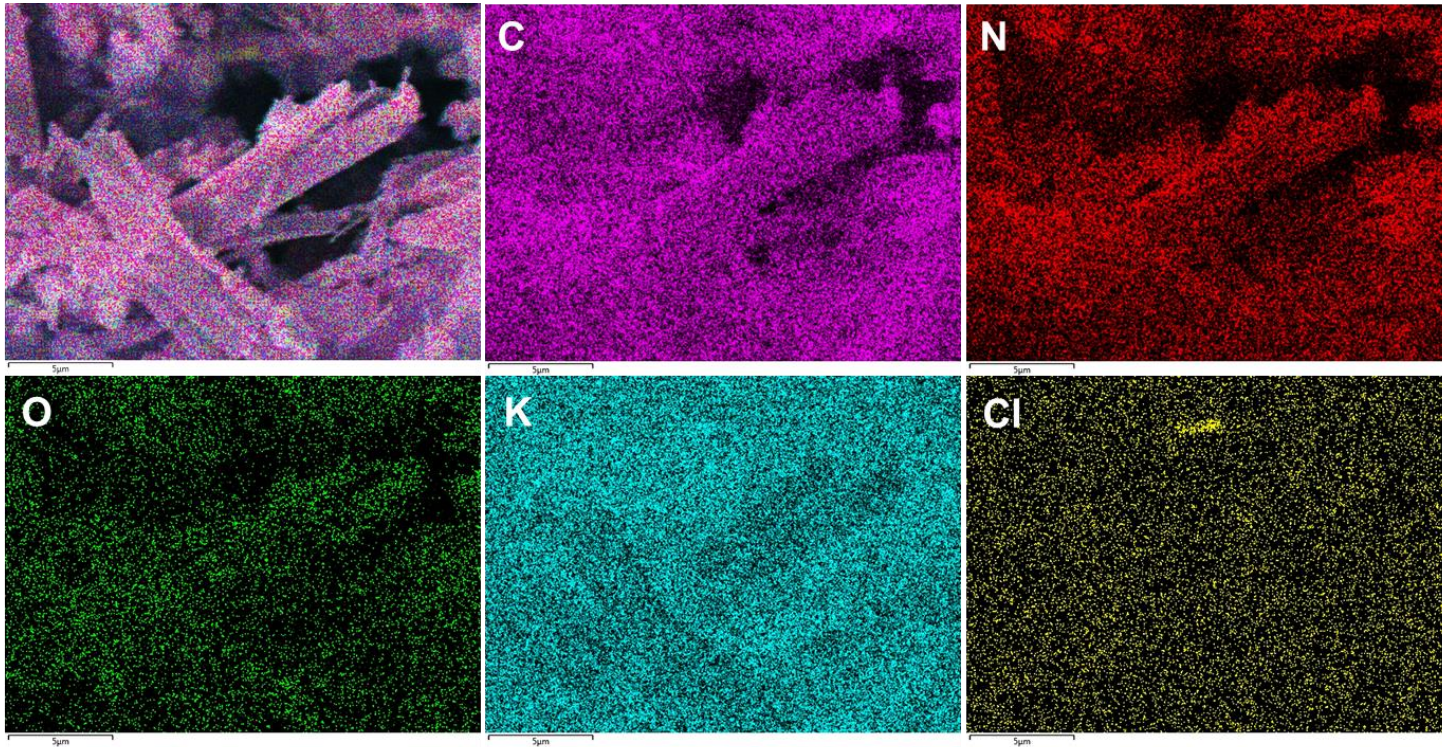

Figure S4. XEDS overlay image and corresponding elemental maps of C, N, O, K, and Cl for a selected area of MX→PHI_{500} sample highlighting the elemental distribution across the thin hollow fibres/rods remains the same even at 500 °C of ionothermal polymerization.

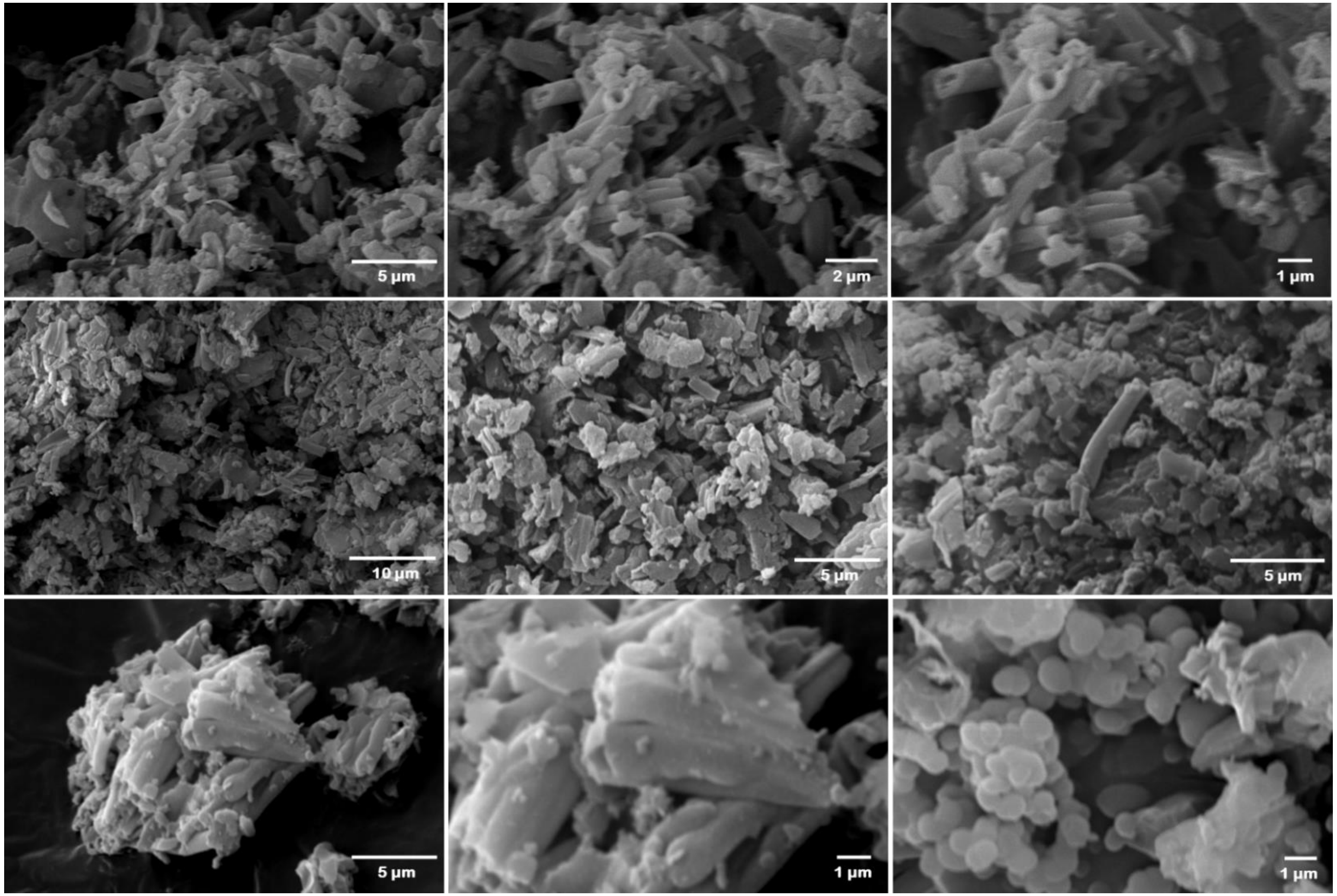

Figure S5. SEM micrographs taken at different magnifications and areas of final MX→PHI photocatalyst. The morphology of these particles is similar to that of MX→PHI_{500} sample (hollow fibres/rods).

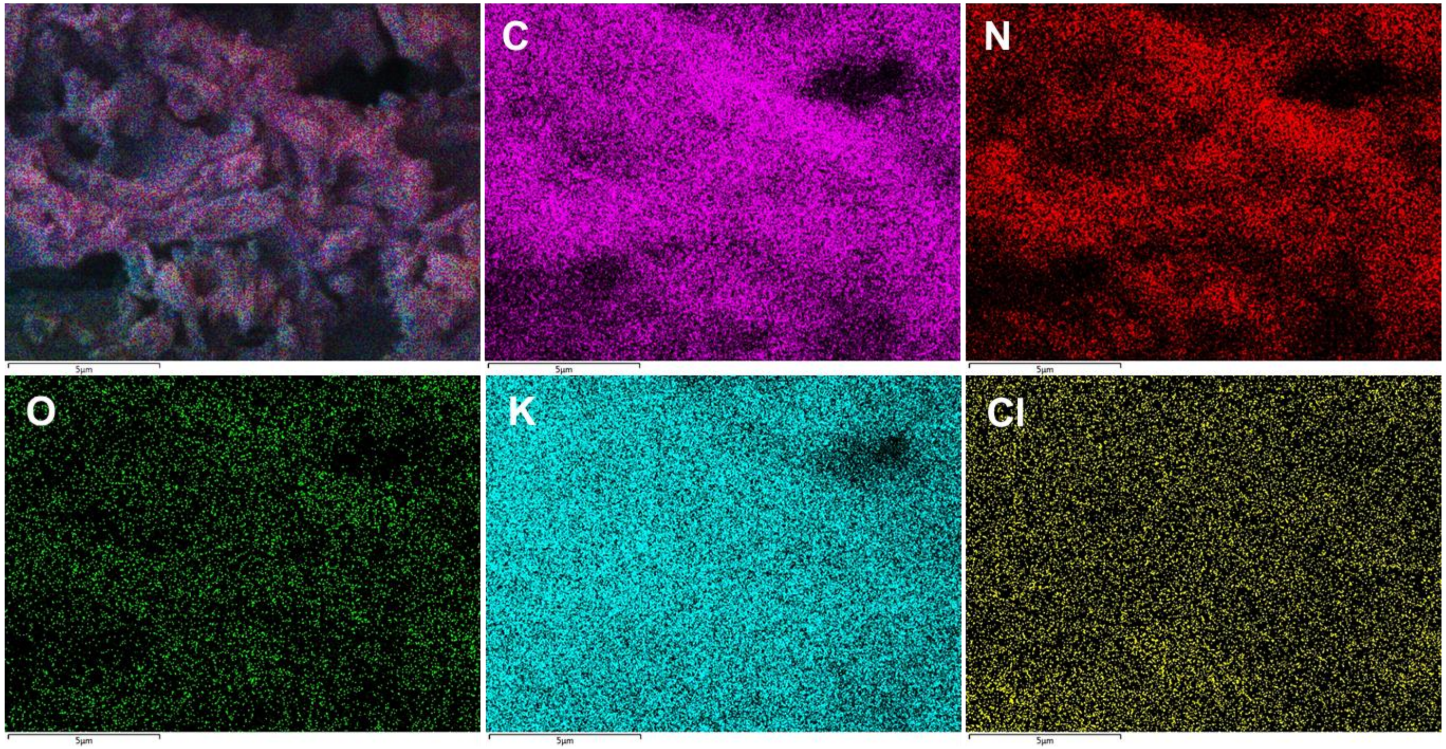

Figure S6. XEDS overlay image and corresponding elemental maps of C, N, O, K, and Cl for selected area of MX→PHI photocatalyst highlight the uniform elemental distribution across the thin hollow fibres/rods.

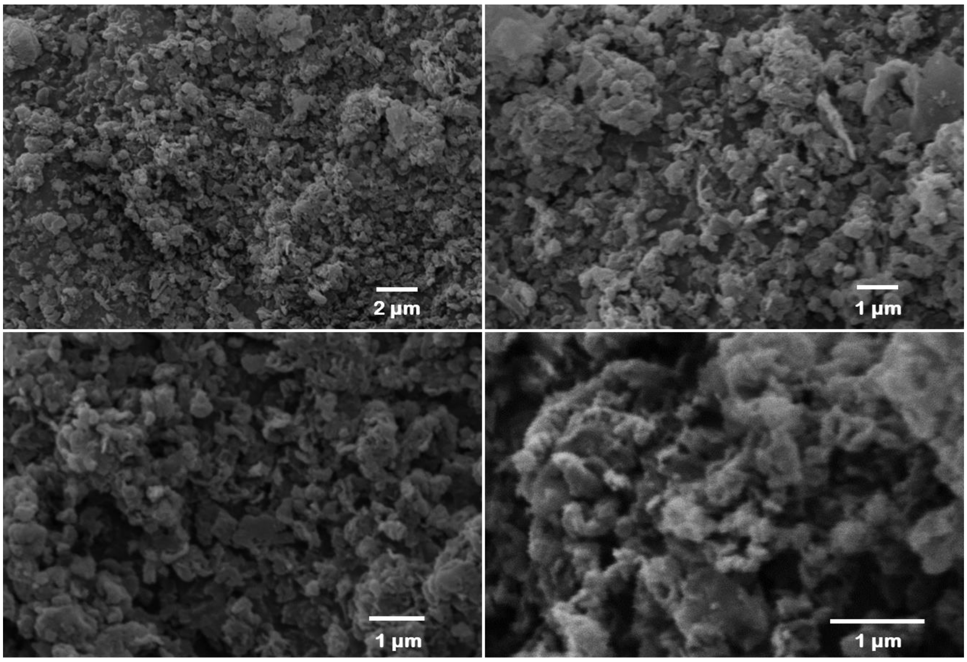

Figure S7. SEM micrographs of as-synthesized BCN sample.

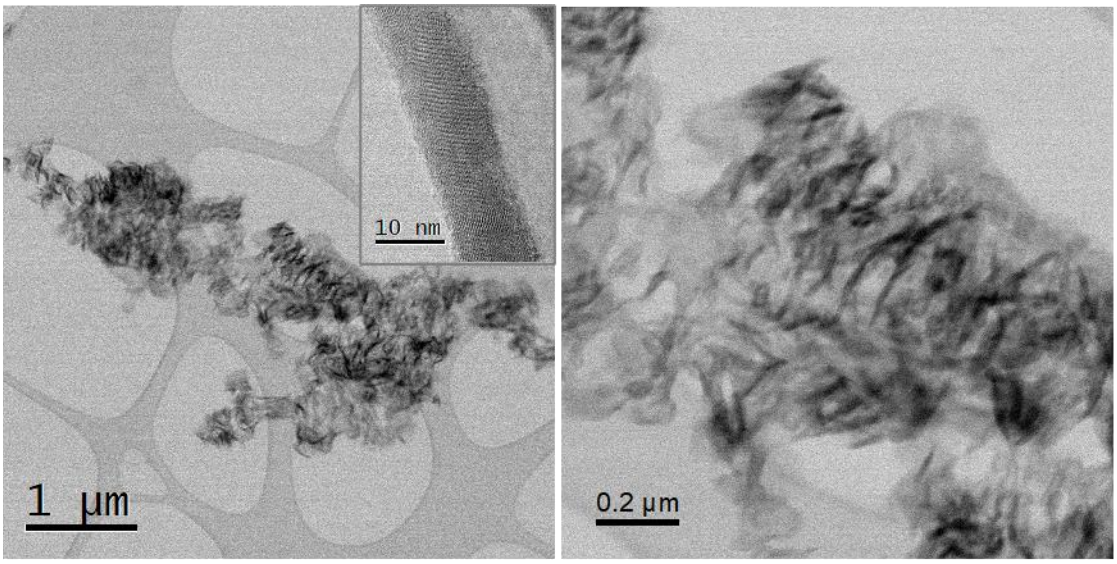

Figure S8. TEM and HRTEM (inset) images highlight the curled-edge sheet morphology of as-synthesized BCN particles.

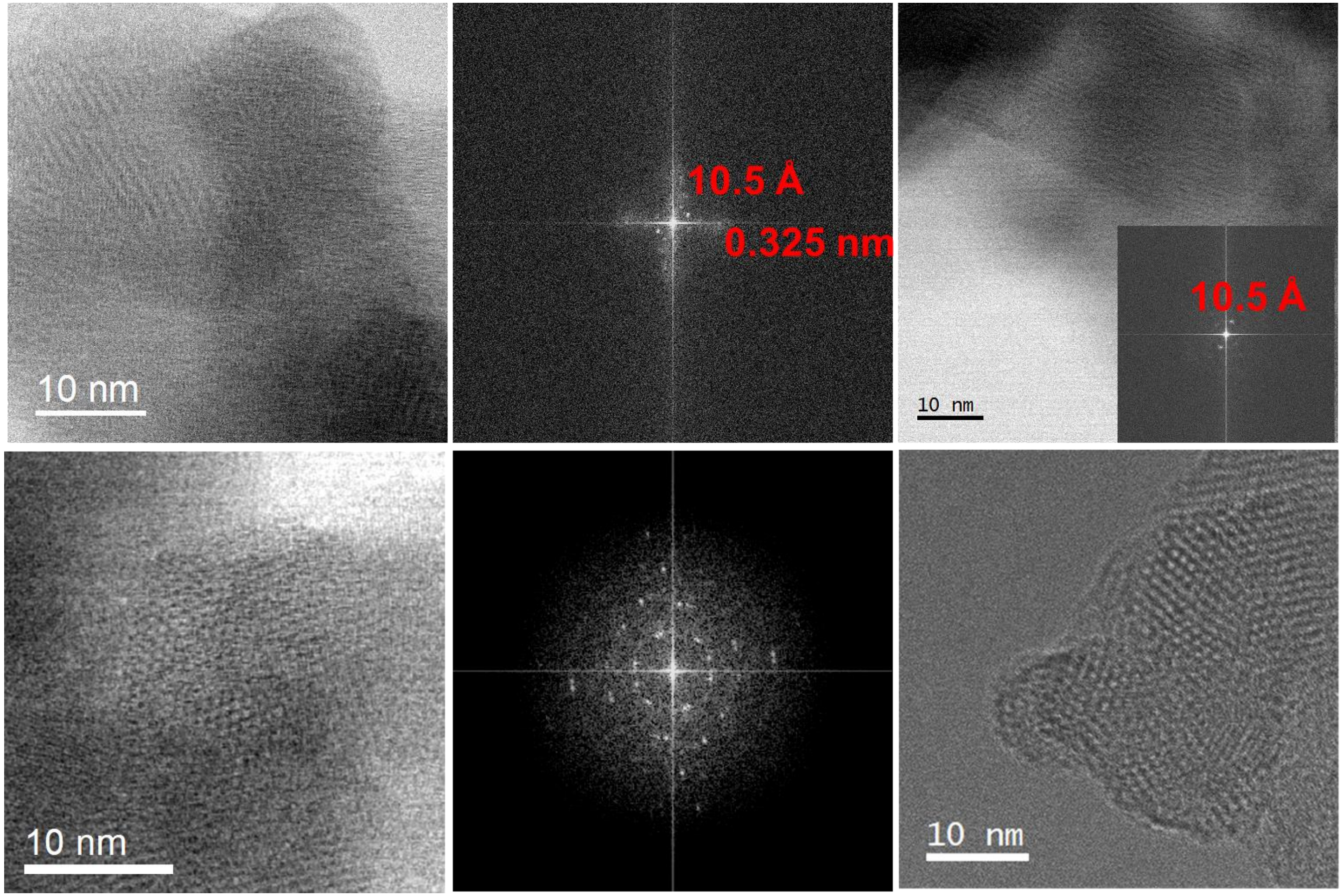

Figure S9. Additional HRTEM images and corresponding FFTs of MX→PHI particles. All these images highlight 1 nm in-plane repeated unit structural packing.

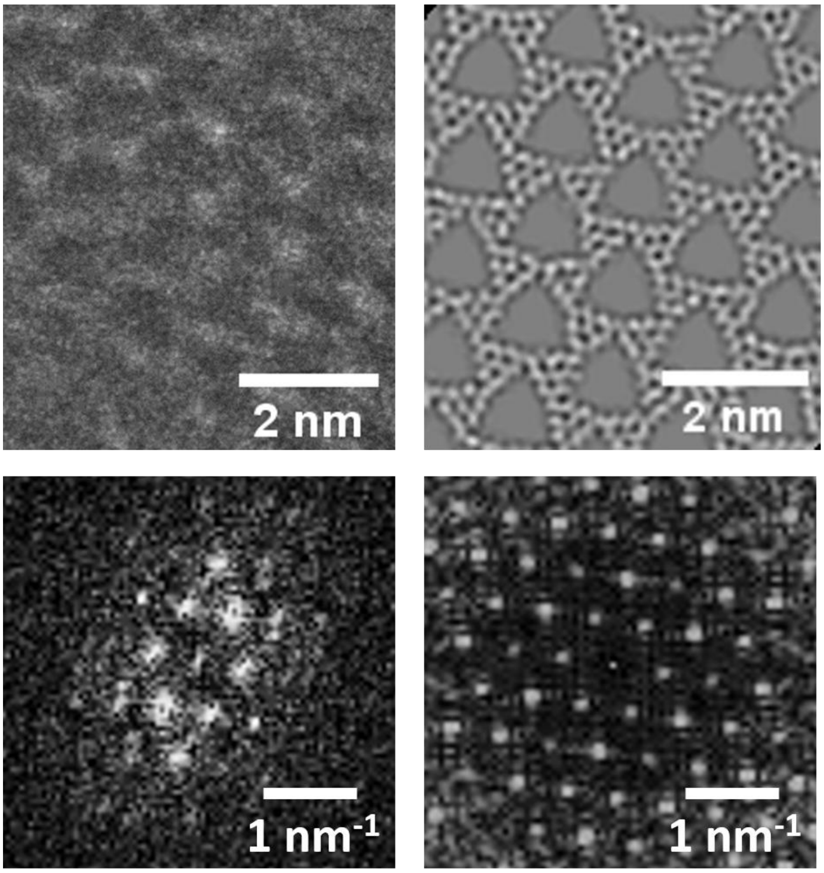

Figure S10. (Left) Region of HRTEM image taken from **Figure 1e**, with corresponding FFT below. (Right) HRTEM simulation, with corresponding FFT below.

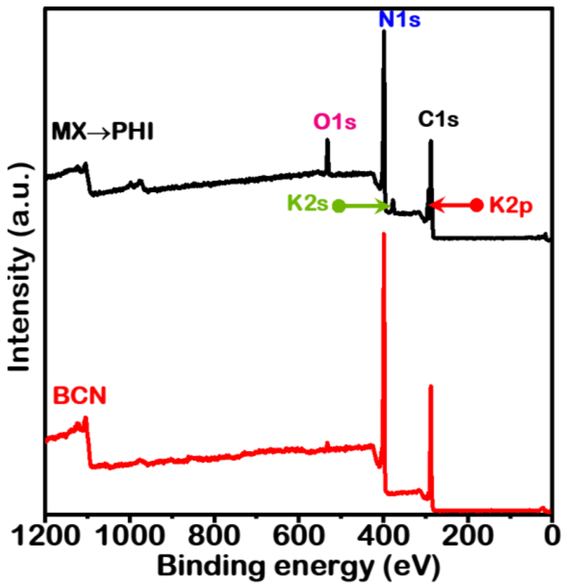

Figure S11. X-ray photoelectron spectroscopy (XPS) survey scan spectra of basic carbon nitride (BCN), and alkali metal halide implanted poly(heptazine imide) molecular photocatalyst (MX→PHI).

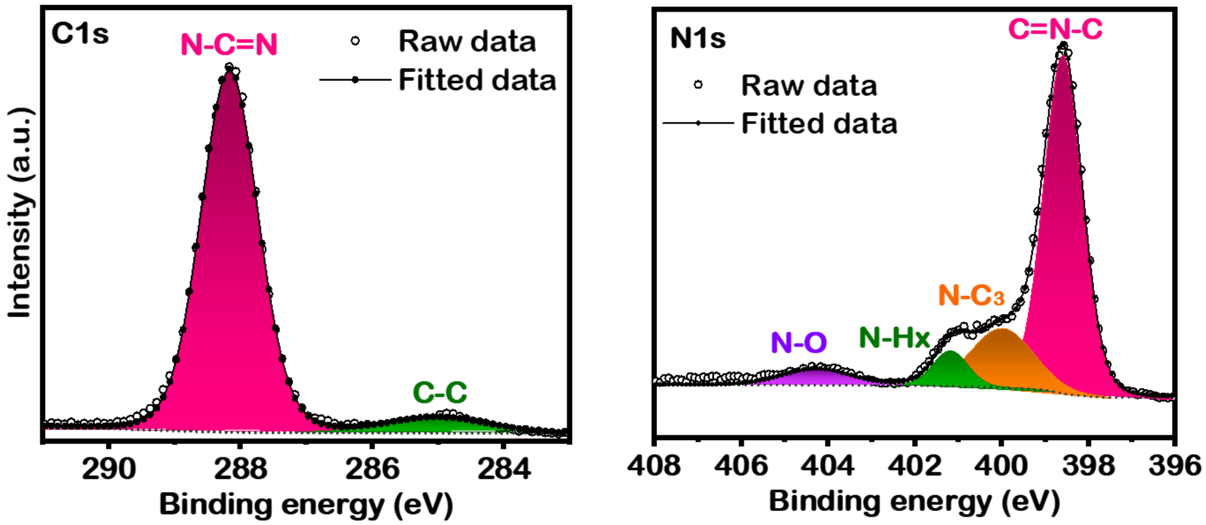

Figure S12. X-ray photoelectron spectroscopy (XPS) core-level spectra for C 1s, and N 1s in BCN photocatalyst.

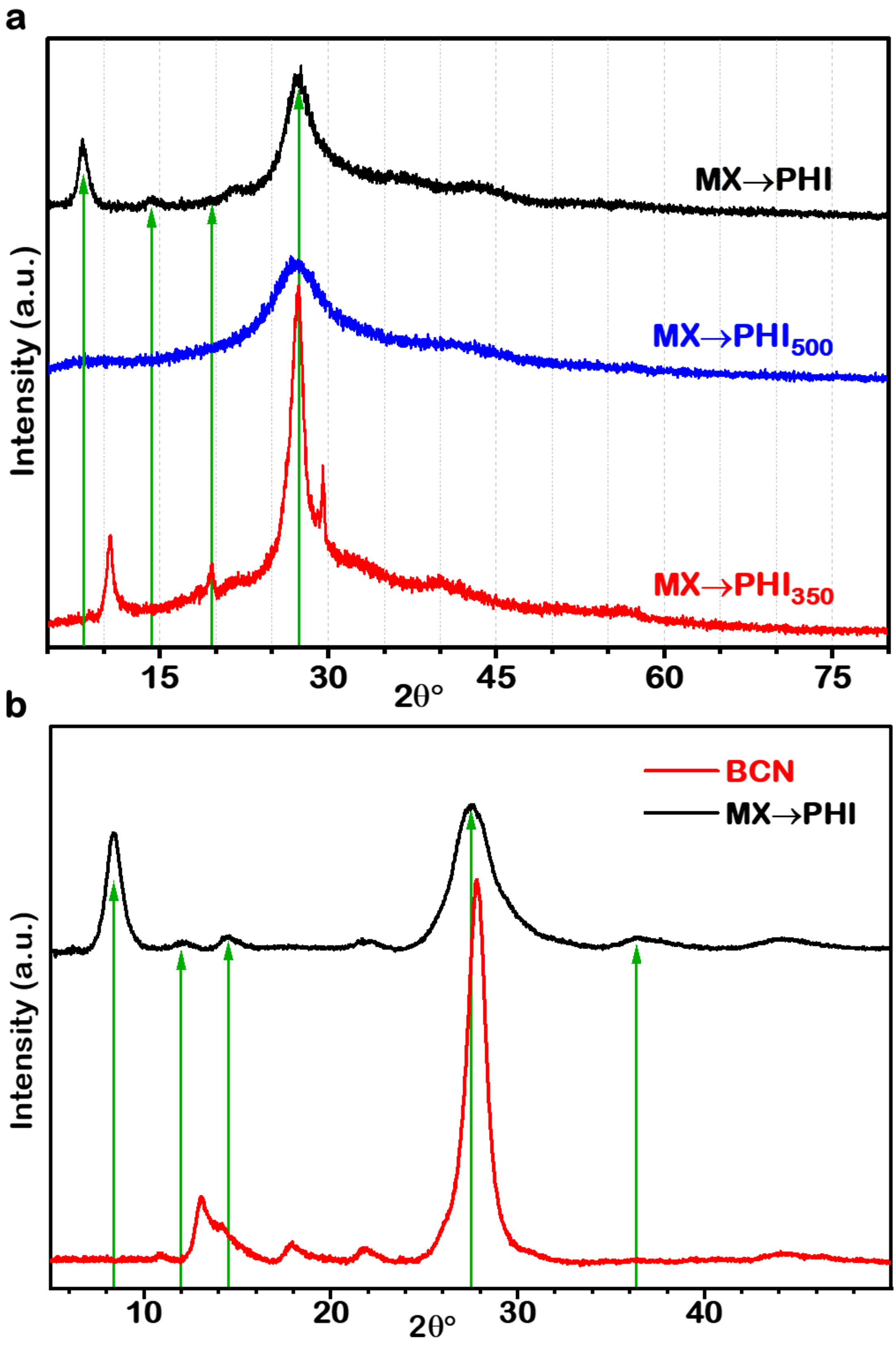

Figure S13. a, XRD pattern of MX→PHI_{350}, MX→PHI_{500}, and MX→PHI samples highlighting the transformation of urea to PHI structure. **b,** Slow scan XRD patterns of BCN and MX→PHI highlight the peak shifts and a comparison among these photocatalysts.

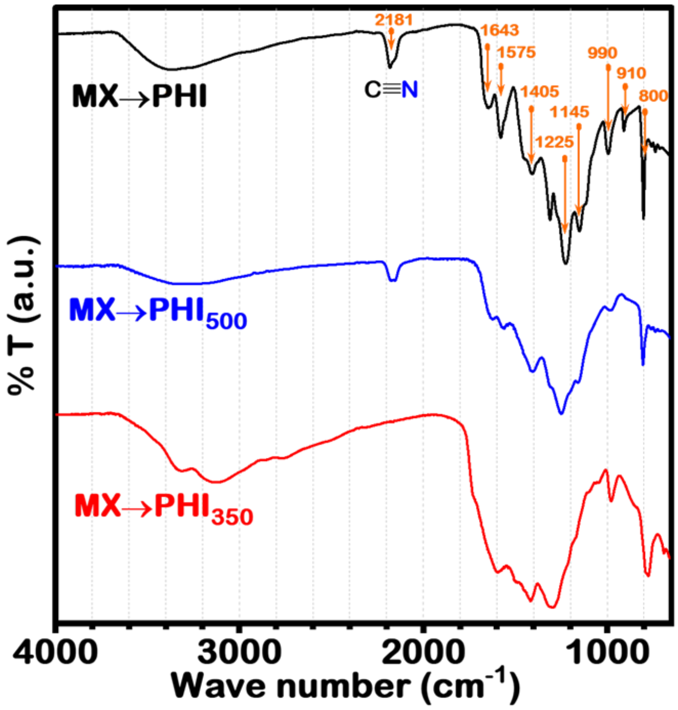

Figure S14. FTIR spectra for MX→PHI350, MX→PHI500, and MX→PHI samples highlighting the polymeric growth of urea to PHI.

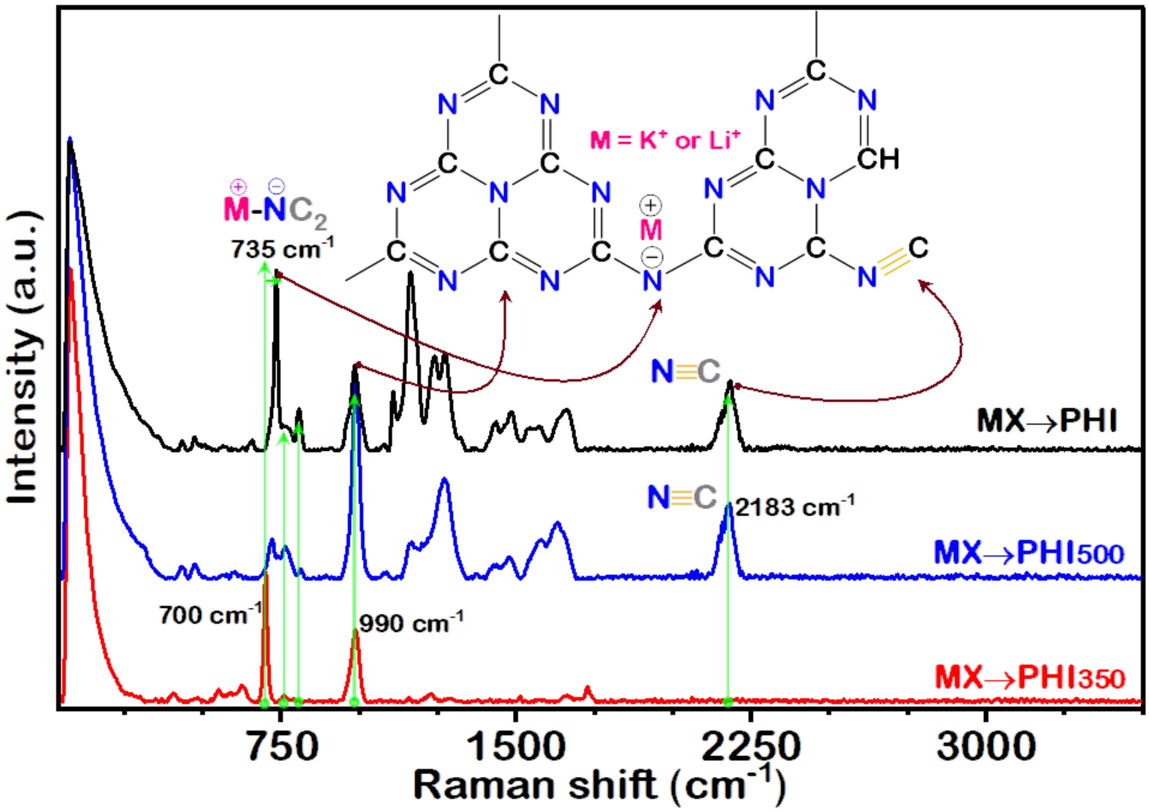

Figure S15. Raman spectra for MX→PHI350, MX→PHI500, and MX→PHI samples recorded on an FT-MultiRam (Bruker) Raman spectrometer equipped with Nd: YAG laser source of excitation 1064 nm to highlight the polymeric growth of MX→PHI photocatalyst. The appearance of sharp peaks at 700, 760, and 990 cm^{-1} in the MX→PHI350 sample correspond to in-plane bending vibrations of the C−N=C and breathing mode of the heptazine ring. These Raman peaks asserted the initialization of C-N-based polymer formation having heptazine as repeated units. However, with the temperature rise, a peak shift of about 35 cm^{-1} to a higher wavenumber was observed for the peak 700 cm^{-1}. As shown in the above spectra this peak shift is probably due to the ionic interaction of positively charged alkali metals (K^+ and Li^+) with the negatively charged nitrogen of PHI units. Further, a strong peak at 2183 cm^{-1} for MX→PHI500, and MX→PHI samples indicates that the as-prepared photocatalyst has been decorated with a cyano group (C≡N).

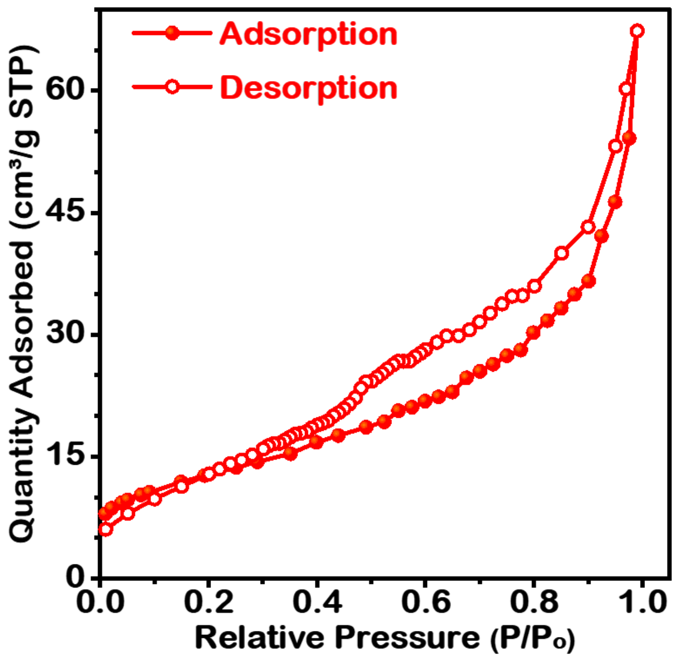

Figure S16. N_2 adsorption-desorption isotherms of BCN.

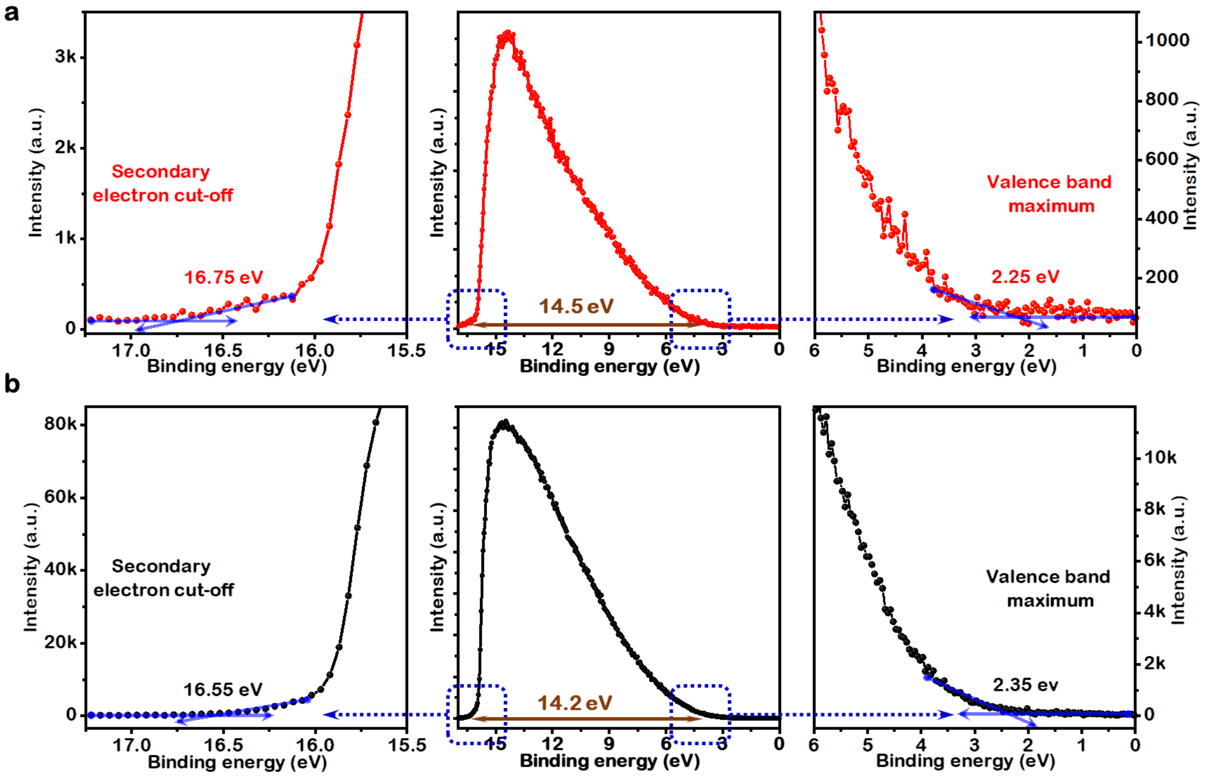

Figure S17. UPS spectra (centre), magnified view of the secondary electron cut-off region (left-hand side), and valence band edges (right-hand side) of as-synthesized BCN (**a**) and MX→PHI (**b**). The valence band maximum for the respective photocatalysts was calculated by estimating the ionization potential (equivalent to the valence band energy) by subtracting the width of the He I UPS spectrum from the excitation energy (21.22 eV). The intersection points of the tangents (downward arrow) with the baseline (horizontal arrow) provide the edge values from which the UPS width is determined.

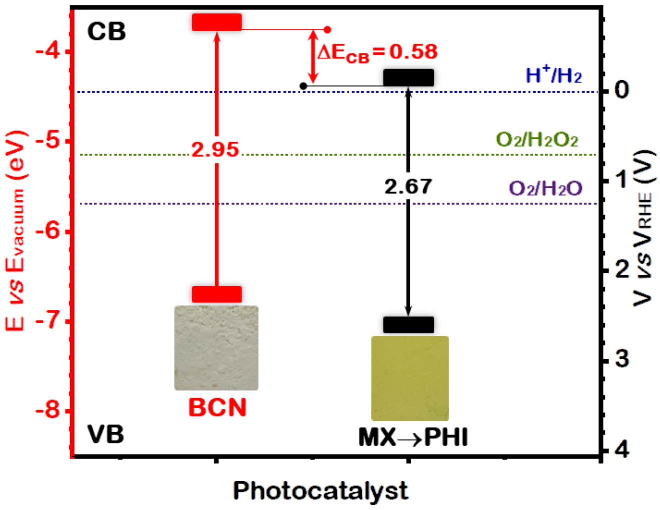

Figure S18. Schematic of bandgap and energy level bands with respect to vacuum level as well as reversible hydrogen electrode (RHE). Inset digital photographs show the colour of the respective photocatalyst.

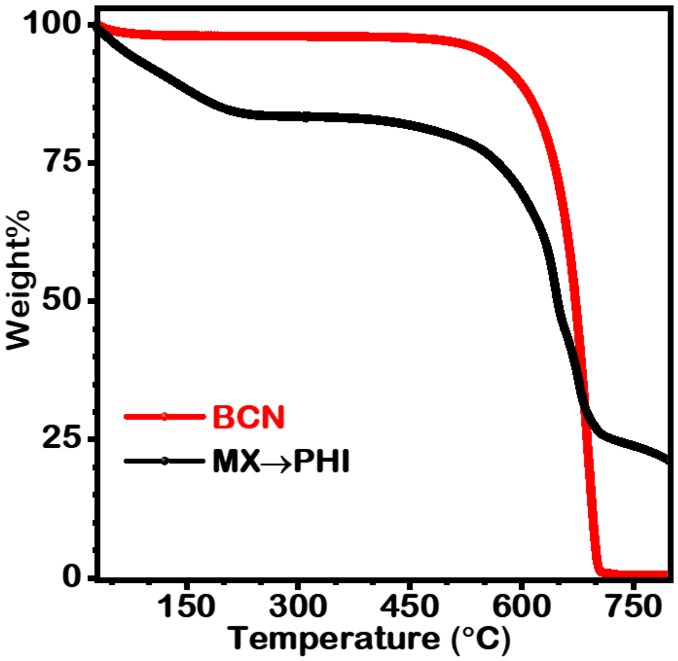

Figure S19. TGA thermograms of BCN, and MX→PHI photocatalysts.

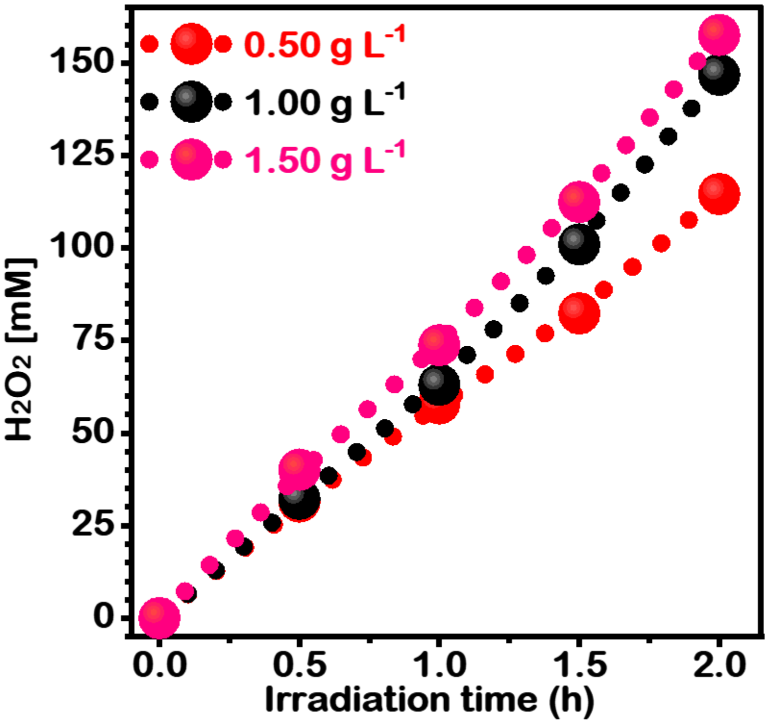

Figure S20. Photochemical H_2O_2 production as a function photocatalyst concentration over porous MX→PHI photocatalyst. Comparison of reductive solar H_2O_2 production from O_2 in 10 M ethanol solution as an electron and proton donor at varying photocatalyst suspension.

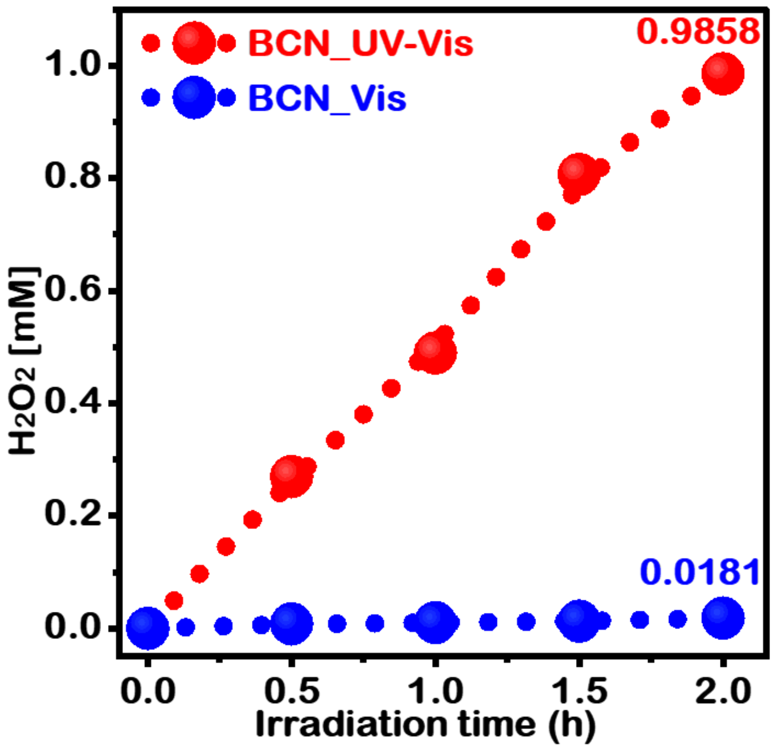

Figure S21. Photochemical H_2O_2 production performance of BCN. Magnified view of H_2O_2 production over time on BCN under UV-visible, and visible (≥ 400 nm) light irradiation reported in **Figure 4d**.

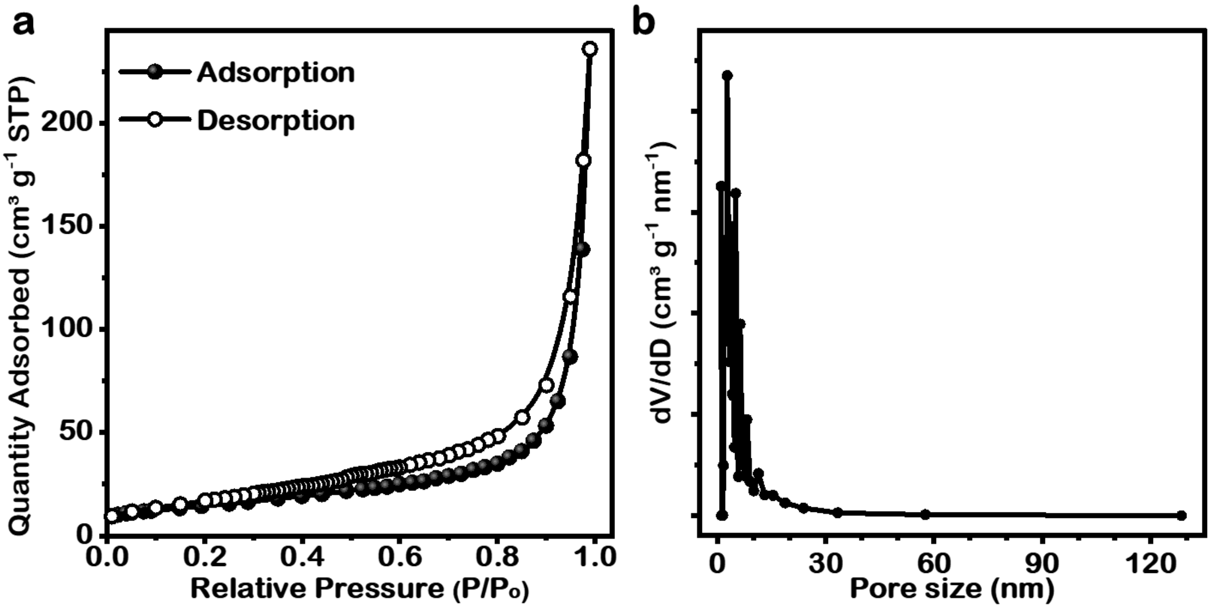

Figure S22. N_2 adsorption-desorption isotherm **(a)**, and Barrett-Joyner-Halenda (BJH) pore size distribution plot **(b)** of MX→PHI sample collected after 1st run (MX→PHI_1^{st} run). Similar to the original MX→PHI material, the MX→PHI_1^{st} run sample also reports a type IV isotherm with an H3 hysteresis loop substantiating that even after the photochemical reaction the material retains its mesoporous character.

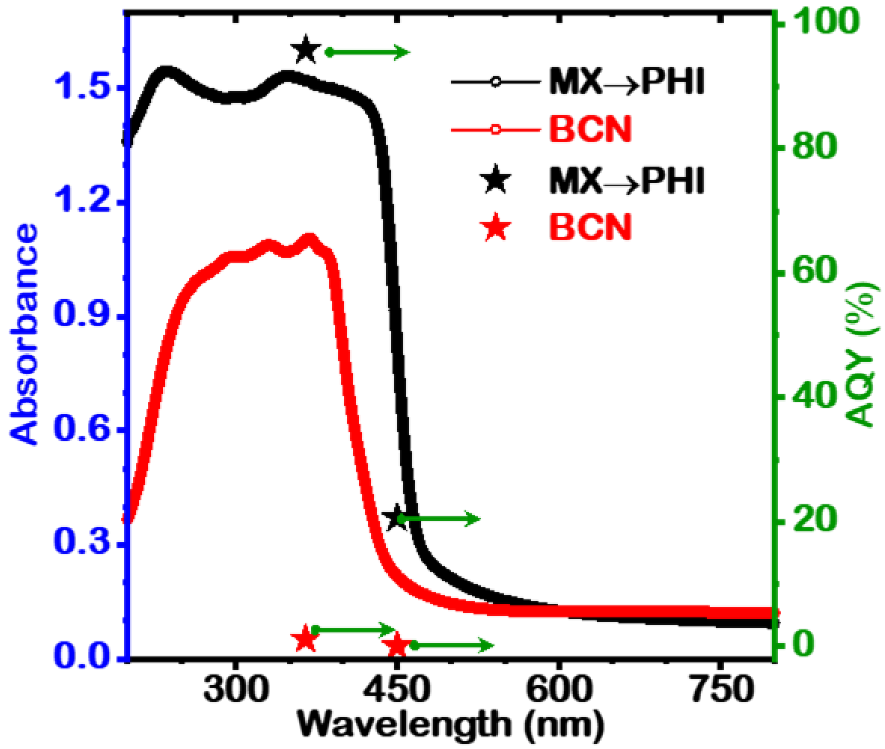

Figure S23. Wavelength-dependent AQY for solar H_2O_2 production over BCN (red stars) and MX→PHI (black stars).

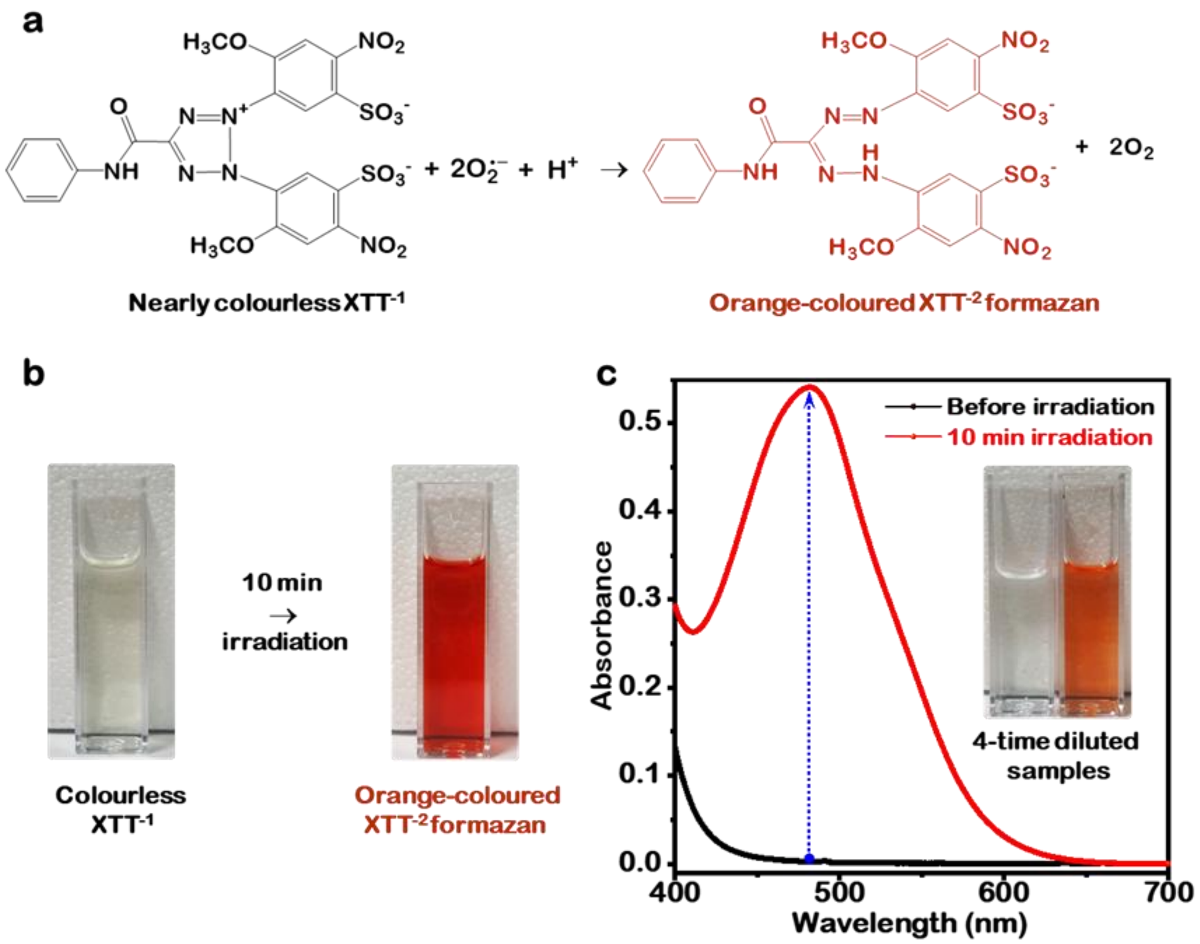

Figure S24. Detection of $O_2^{\bullet-}$ intermediate reaction species generated during photochemical H_2O_2 production over MX→PHI photocatalyst. a, The chemical reaction shows the reaction of colourless XTT with $O_2^{\bullet-}$ to produce orange coloured XTT-formazan. **b,** Optical images highlight the transformation of colourless XTT sodium salt solution to orange coloured XTT-formazan on 10 min UV-visible light irradiation of MX→PHI suspension (concentration 0.1 g L^{-1}) in 1 mM XTT solution (in 10 M ethanol). **c,** UV−vis absorption spectra of aliquots collected from MX→PHI suspension in 1 mM XTT solution before light irradiation and after 10 min of irradiation. The inset is a digital photo of the quartz vials containing diluted XTT solutions used for spectrophotometric analysis.

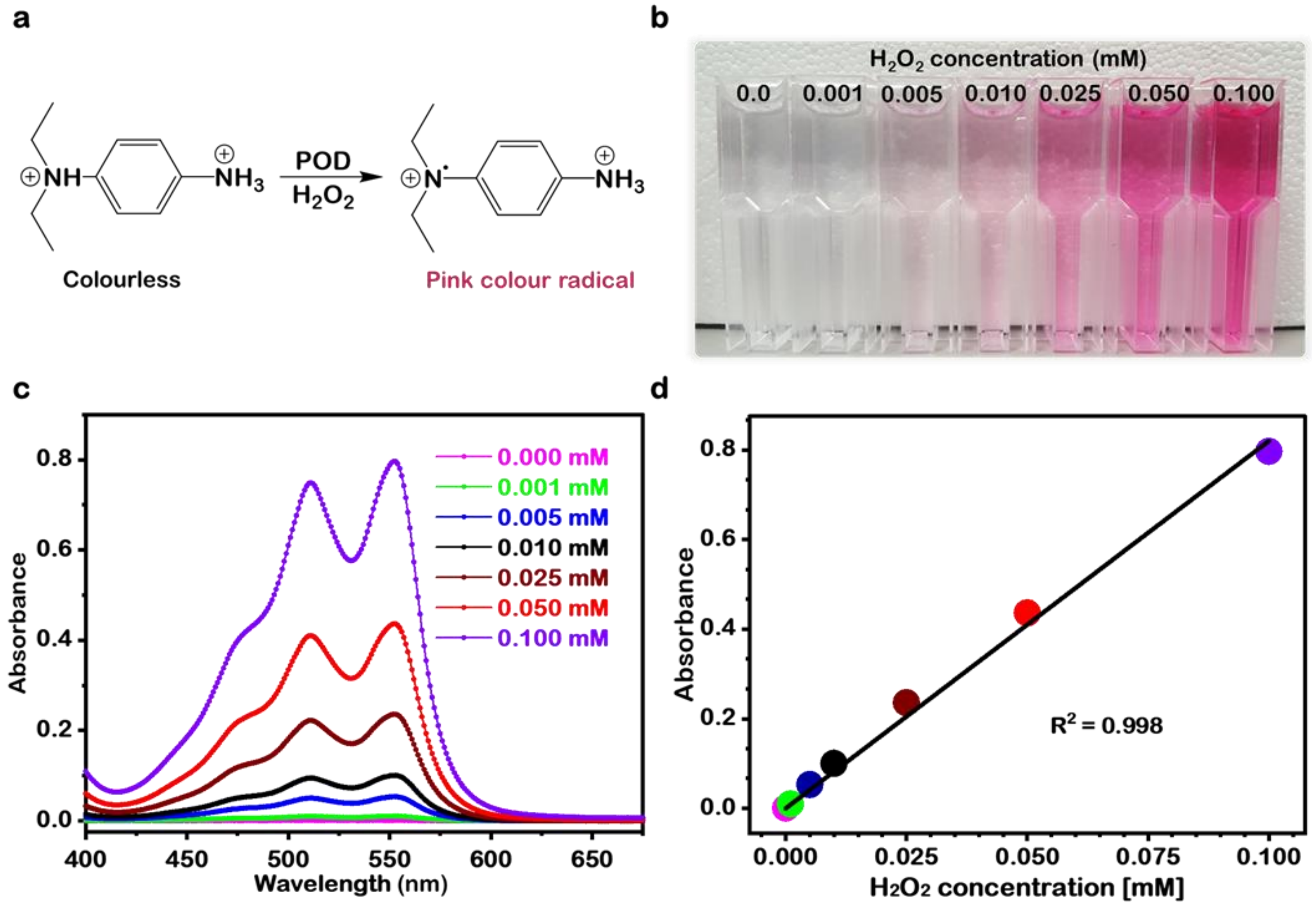

Figure S25. Spectrophotometric quantification of H_2O_2. a, Reaction pathway of highly visible light absorbing N,N-diethyl-p-phenylenediamine (DPD) radical cation formation on DPD oxidation by H_2O_2 in presence of peroxidase (POD)**. b,** Digital photographs of pink colour DPD radical cation formed on the oxidation of DPD in the presence of H_2O_2 and POD. **c,** Absorbance spectrum of DPD radical cation on oxidation by different H_2O_2 concentrations. **d,** Calibration curve for spectrophotometric quantification of photogenerated H_2O_2 content in the reaction solution.

Table S1. Elemental analysis data using XPS, STEM-XEDS, and ICP-MS analyses for MX→PHI sample.

Element	Analysis method		
	XPS (at.%)	STEM-XEDS (at.%)	ICP-MS (wt.%)[a]
C	36.83	54.6 ± 5.5	-
N	50.69	38.6 ± 3.9	-
K	1.86	4.9 ± 0.5	1.97
Li	2.80	-	1.59
Cl	0.83	0.9 ± 0.1	-
O	6.99	1.0 ± 0.1	-

[a]*The high background of O and Cl leads to interference.*

Table S2. Comparison of photocatalysts for solar H_2O_2 production from oxygen saturated solution of electron and proton donor solvents. Operation condition includes solvent used, concentration of solvent, light source, maximum reported accumulated H_2O_2 concentration corresponding to total time of light irradiation. Solar H_2O_2 production rate has been calculated using maximum reported accumulated H_2O_2 concentration with respect to total time of irradiation.

	Photocatalyst	Reaction conditions	Optical irradiance[a] (mW cm^{-2})	H_2O_2 production			Ref.
				Conc.[n] [mM]	Time (h)	Rate (mM h^{-1})	
Carbon nitride	g-C_3N_4	90% (v) 2-propanol/water (5 mL); 4 g L^{-1}; 2000 W Xe lamp (≥420 nm)	2.69 (420–500 nm)	12.00	12	1.00	6
	g-C_3N_4/PDI	90% (v) 2-propanol/water (30 mL); 1.7 g L^{-1}; 2000W Xe lamp (≥420 nm)	2.69 (420–500 nm)	7.00	6	1.17	7
	g-C_3N_4_SiO2 tempted	90% (v) ethanol/water (5 mL); 4 g L^{-1}; 2000 W Xe lamp (≥420 nm)	2.69 (420–500 nm)	18.20	24	0.76	8
	g-C_3N_4_Phototreated hydrazine	20% (v) 2-propanol/water (60 mL); 0.83 g L^{-1}; solar simulator (≥420 nm)	-	0.20	2.5	0.08	9
	N vacancy g-C_3N_4	50% (v) ethanol/water (200 mL); 1.0 g L^{-1}; 250 W high-pressure Na lamp (≥ 400 nm)	-	5.20	18	0.29	10
	g-C_3N_4-Carbon	5% (v) 2-propanol/water; pH3, 1.0 g L^{-1}; 300W Xe lamp	100.00	1.27	4	0.32	11
	g-C_3N_4-CNT	5% (v) formic acid/water (100 mL); 1.0 g L^{-1}; 300W Xe lamp (≥ 400 nm)	-	0.33	1	0.33	12
	AQ-augmented g-C_3N_4	10% (v) 2-propanol/water; 0.5 g L-1; AM 1.5	100.00	0.94	3	0.31	13
	Benzene substituted g-C_3N_4	10% (v) ethanol/water (30 mL); pH3, 0.5 g L^{-1}; 300W Xe lamp (≥420 nm)	-	0.30	3	0.10	14
	g-C_3N_4-NDCN	30% (v) 2-propanol/water (100 mL); 0.5 g L^{-1}; solar simulator (≥ 420 nm)	-	2.40	10	0.24	15
	Phosphate/g-C_3N_4	2.6 mM EDTA solution (200 mL); 1.0 g L^{-1}; 250W high-pressure Na lamp (≥ 400 nm)	-	5.40	50	0.11	16
	g-C_3N_4-BDI	90% (v) 2-propanol/water (30 mL); 3.3 g L^{-1}; solar simulator (≥ 420 nm)	2.73 (420–500 nm)	1.48	2	0.74	17
	g-C_3N_4-MTI	90% (v) 2-propanol/water (30 mL); 1.7 g L^{-1}; solar simulator Xe lamp (≥ 420 nm)	100.00	1.55	2	0.78	18

	$g-C_3N_4$-PDI-rGO	90% (v) 2-propanol/water (30 mL); 1.7 g L^{-1}; Xe lamp (≥ 420 nm)	-	20.00	9	2.22	[19]
	$g-C_3N_4$/PDI-BN-rGO	90% (v) 2-propanol/water (30 mL); 1.7 g L^{-1}; Xe lamp (≥ 420 nm)	4.3 (420–500 nm)	18.33	6	3.06	[20]
	BP/$g-C_3N_4$	10% (v) 2-propanol/water (30 mL); 1.7 g L^{-1}; 300W Xe lamp (≥ 420 nm)	-	4.90	12	0.41	[21]
	BNQDs/UP $g-C_3N_4$	10% (v) 2-propanol/water (50 mL); 1.0 g L^{-1}; 300W Xe lamp (≥ 420 nm)	-	0.07	1	0.07	[22]
	$g-C_3N_4$/hBN	5% (v) methanol/water (40 mL); pH3; 0.5 g L^{-1}; 300 W Xe lamp (≥ 305 nm)	-	2.88	4	0.72	[23]
	(K, P, and O)/$g-C_3N_4$	10% (v) ethanol/water (50 mL); pH 3; 0.5 g L^{-1}; 300W Xe lamp (≥ 420 nm)	726.8	1.70	7	0.24	[24]
	CoP/$g-C_3N_4$	10% (v) ethanol/water (20 mL); 1.0 g L^{-1}; 300W Xe lamp (≥ 420 nm)	-	0.140	2	0.07	[25]
	Au/$g-C_3N_4$	5% (v) 2-propanol/water; pH 3; 1.0 g L^{-1}; Xe lamp	100.00	1.32	4	0.33	[26]
	Cu/$g-C_3N_4$	1 mM EDTA-2Na (200 mL); 1.0 g L^{-1}; 250W high-pressure Na lamp (400-800 nm)	-	7.8	18	0.43	[27]
	KPF_6/$g-C_3N_4$	10% (v) ethanol/water; pH 3; 0.5 g L^{-1}; 300W Xe lamp (≥ 420 nm)	-	1.51	5	0.30	[28]
	N vacancy $g-C_3N_4$	0.789 g L^{-1} ethanol (200 mL); 1.0 g L^{-1}; 250W high-pressure Na lamp (400-800 nm)	-	4.40	18	0.24	[29]
	$Cu_2(OH)_2CO_3$/ $g-C_3N_4$	EDTA-2Na solution (200 mL); 1.0 g L^{-1}; 300 W Xe lamp and 200 W infrared light	-	12.1	18	0.67	[30]
	K^+, Na^+/$g-C_3N_4$	EDTA-2Na (200 mL); 1.0 g L-1; 250W high-pressure Na lamp (400-800 nm)	-	3.1	18	0.17	[31]
	O-enriched C_3N_4 polymer	10% (v) 2-propanol/water (50 mL); 1.0 g L^{-1}; 300 W Xe lamp (≥ 420 nm)	35.2	14.6	5	2.92	[32]
	Br-H/$g-C_3N_4$	EDTA-2Na (200 mL); 1.0 g L-1; 250W high-pressure Na lamp (400-800 nm)	-	1.99	50	0.04	[33]
	SiW_{11}/$g-C_3N_4$	5% (v) methanol/water (100 mL); 1 g L^{-1}; 300W Xe lamp AM 1.5 filter	-	0.18	1	0.18	[34]
	K_2HPO_4/$g-C_3N_4$	10% (v) ethanol/water (100 mL); 1.0 g L^{-1}; 300W Xe lamp (≥ 420 nm)	300.00	5.05	10	0.51	[35]
	$g-C_3N_4$-CoWO	Ethanol (100 mL); 1.0 g L^{-1}; 300W Xe lamp (≥ 420 nm)	-	0.19	1	0.19	[36]
	Au/$g-C_3N_4$	10% (v) ethanol/water (100 mL); pH 8.5; 4.0 g L^{-1}; 300W Xe lamp (≥ 420 nm)	-	2.03	30	0.07	[37]
	Ti_3C_2/$g-C_3N_4$	10% (v) 2-propanol/water (50 mL); 1.0 g L^{-1}; 300W Xe lamp (≥ 420 nm)	-	0.13	1	0.13	[38]

	$KCl/g\text{-}C_3N_4$	10% (v) ethanol/water (50 mL); pH 3; 0.5 g L^{-1}; 300 W Xe lamp (≥ 420 nm)	-	3.4	3	1.13	39
Metal oxides	Ag/TiO_2	5% (v) 2-propanol/0.1 M phosphate buffer (40 mL); pH 3; 0.5 g L^{-1}; 300W Xe lamp (≥ 320 nm)	-	5.9	3	1.97	40
	Au/TiO_2	5% (v) 2-propanol/0.1 M phosphate buffer (40 mL); pH 3; 0.5 g L^{-1}; 300W Xe lamp (≥ 320 nm)	-	5.2	3	1.73	40
	rGO/TiO_2	5% (v) 2-propanol/0.1 M phosphate buffer (40 mL); pH 3; 0.5 g L^{-1}; 300W Xe lamp (≥ 320 nm)	-	4.5	3	1.50	40
	$F\text{-}TiO_2$	10 mM Formic acid, 10 mM [HF] + [F^-] (5 mL); pH 3.2; 0.5 g L^{-1}; 40 W fluorescent lamp; Air	-	1.3	2	0.65	41
	Au/TiO_2	4% (v) ethanol/water (200 mL); 1 g L^{-1}; UV light (≥ 300 nm)	3.00 (290–390 nm)	10	24	0.42	42
	$AuAg/TiO_2$	4% (v) ethanol/water (5 mL); 1 g L^{-1}; 450 W high pressure Hg lamp (≥ 280 nm)	13.80 (280–400 nm)	3.4	12	0.28	43
	TiO_2	0.35 M 1-propanol (5 mL); 10 g L^{-1}; 450 W high pressure Hg lamp (≥ 280 nm)	13.80 (280–400 nm)	39.6	12	3.30	44
	Au/TiO_2	4% (v) ethanol/water (200 mL); pH 1.95; 1 g L^{-1}; high pressure Hg lamp (≥ 300 nm)	3.00 (290–390 nm)	14.0	24	0.58	45
	$S,N\text{-}GQD/TiO_2$	6% (v) 2-propanol/water (50 mL); pH 3; 0.5 g L^{-1}; 500W Xe lamp (≥ 300 nm)	-	0.45	1	0.45	46
	Au/TiO_2	4% (v) methanol/2 mM phosphate (50 mL); pH 8; 1 g L^{-1}; high pressure Hg lamp (≥ 320 nm)	4.50	1.35	10	0.14	47
	Co/TiO_2	Methanol + 0.1 M potassium hydrogen phthalate (50 mL); pH 3.9; 1 g L^{-1}; LED (400 nm)	-	0.5	1	0.50	48
	$Au@SnO_2/TiO_2$	4% alcohol/water (200 mL); 1.0 g L^{-1}; 300 W Xenon lamp	-	15.5	26	0.60	49
	Au/WO_3	90% (v) ethanol/water (30 mL); 1.7 g L^{-1}; 2 kW Xe lamp (≥ 420 nm)	2.69 (420–500 nm)	0.29	10	0.03	50
	$Au\ /BiVO_4$	90% (v) ethanol/water (30 mL); 1.7 g L^{-1}; 2 kW Xe lamp (≥ 420 nm)	2.69 (420–500 nm)	0.26	10	0.03	50
	$Bi/Bi_2O_{2-x}CO_3$	Ethanol/water (30 mL); 300 W Xenon lamp (≥ 420 nm)	-	1.21	3	0.40	51
Metal sulphides	S-doped Carbon/CdS	20% (v) 2-propanol/KOH solution (25 mL); pH 14; 1.0 g L^{-1}; 300W Xe lamp (≥ 420 nm)	-	17.1	24	0.71	52
	S-doped stable Carbon/CdS	20% (v) 2-propanol/KOH solution (25 mL); pH 14; 1.0 g L^{-1}; 300W Xe lamp (≥ 420 nm)	-	11.6	24	0.48	52
	GOND/CdS-SNC	5% (v) methanol/water; pH 4; 4.0 g L^{-1}; LED (2400 mW cm^{-2}, 635 nm)	2400	0.10	1	0.10	53
	Au/MoS_2	25% (v) ethanol/water (50 mL); pH 9; 1.0 g L^{-1}; 10 °C; 300W Xe lamp	-	0.98	12	0.08	54

Organic/Organometallic	RF-resin	0.35 M Benzyl alcohol (30 mL); 1.7 g L^{-1}; 2000W Xe lamp (≥420 nm)	14.03 (420–700 nm)	1.43	6	0.24	55
	MIL-125-NH_2	71% (v) Benzyl alcohol/water (7 mL); 0.7 g L^{-1}; 500W Xe lamp (≥420 nm)	-	2.4	3	0.80	56
	Ni/MIL-125-NH_2	Acetonitrile solution (10 mL) of benzyl alcohol (2 mL); 1.0 g L^{-1}; 500W Xe lamp (≥420 nm)	-	8.0	8	1.00	57
	CD/WPU	Ethanol; Normal sunlight	-	0.08	50	0.00	58
	$QuPh^+$-NA	20% MeCN-H_2O, 50 mM oxalic acid, 0.1M TBA acetate, Xe lamp (≥340 nm)	-	35.0	5	7.00	59
	$Cd_3(C_3N_3S_3)_2$	5% (v) methanol/water (20 mL); pH 2.8; 4.0 g L^{-1}; 300W Xe lamp (≥420 nm); Air	-	8.75	4	2.19	60
	TF_{50}-COF	10% (v) ethanol/water (50 mL); 0.1 g L^{-1}; 300W Xe lamp	-	0.28	1	0.28	61
	COF-TTA-TTTA	10% (v) ethanol/water; Blue LEDs (420 nm) 50W	100.00	4.35	8	0.54	62
	DE7-M	10% (v) 2-propanol/water (3 mL); 1.7 g L^{-1}; Oriel Solar Simulator 94123A	100.00	5.67	1.5	3.77	63
This work	MX→PHI	10 M ethanol; 1 g L^{-1}; 150W Xe lamp	175.00	146.80	2	73.40	This work
	MX→PHI	10 M ethanol; 1 g L^{-1}; 150W Xe lamp (≥ 400 nm)	175.00	77.3	2	38.7	
	BCN	10 M ethanol; 1 g L^{-1}; 150W Xe lamp	175.00	0.99	2	0.49	
	BCN	10 M ethanol; 1 g L^{-1}; 150W Xe lamp (≥ 400 nm)	175.00	0.02	2	0.01	

[a]Optical irradiance and experimental conditions subjected to verification.

References

(1) Madsen, J.; Susi, T. The AbTEM Code: Transmission Electron Microscopy from First Principles. *Open Res. Eur.* **2021**, *1*, 24. https://doi.org/10.12688/openreseurope.13015.2.

(2) Cui, M.; Cui, K.; Liu, X.; Chen, X.; Chen, Y.; Guo, Z. Roles of Alkali Metal Dopants and Surface Defects on Polymeric Carbon Nitride in Photocatalytic Peroxymonosulfate Activation towards Water Decontamination. *J. Hazard. Mater.* **2022**, *424*, 127292. https://doi.org/10.1016/j.jhazmat.2021.127292.

(3) Li, Y.; Xu, H.; Ouyang, S.; Lu, D.; Wang, X.; Wang, D.; Ye, J. In Situ Surface Alkalinized g-C_3N_4 toward Enhancement of Photocatalytic H_2 Evolution under Visible-Light Irradiation. *J. Mater. Chem. A* **2016**, *4* (8), 2943–2950. https://doi.org/10.1039/c5ta05128b.

(4) Li, Y.; Ouyang, S.; Xu, H.; Wang, X.; Bi, Y.; Zhang, Y.; Ye, J. Constructing Solid-Gas-Interfacial Fenton Reaction over Alkalinized-C_3N_4 Photocatalyst to Achieve Apparent Quantum Yield of 49% at 420 nm. *J. Am. Chem. Soc.* **2016**, *138* (40), 13289–13297. https://doi.org/10.1021/jacs.6b07272.

(5) Xu, W.; An, X.; Zhang, Q.; Li, Z.; Zhang, Q.; Yao, Z.; Wang, X.; Wang, S.; Zheng, J.; Zhang, J.; Wu, W.; Wu, M. Cesium Salts as Mild Chemical Scissors to Trim Carbon Nitride for Photocatalytic H_2 Evolution. *ACS Sustain. Chem. Eng.* **2019**, *7* (14), 12351–12357. https://doi.org/10.1021/acssuschemeng.9b01717.

(6) Shiraishi, Y.; Kanazawa, S.; Sugano, Y.; Tsukamoto, D.; Sakamoto, H.; Ichikawa, S.; Hirai, T. Highly Selective Production of Hydrogen Peroxide on Graphitic Carbon Nitride (g-C_3N_4) Photocatalyst Activated by Visible Light. *ACS Catal.* **2014**, *4* (3), 774–780. https://doi.org/10.1021/cs401208c.

(7) Shiraishi, Y.; Kanazawa, S.; Kofuji, Y.; Sakamoto, H.; Ichikawa, S.; Tanaka, S.; Hirai, T. Sunlight-Driven Hydrogen Peroxide Production from Water and Molecular Oxygen by Metal-Free Photocatalysts. *Angew. Chemie Int. Ed.* **2014**, *53* (49), 13454–13459. https://doi.org/10.1002/anie.201407938.

(8) Shiraishi, Y.; Kofuji, Y.; Sakamoto, H.; Tanaka, S.; Ichikawa, S.; Hirai, T. Effects of Surface Defects on Photocatalytic H_2O_2 Production by Mesoporous Graphitic Carbon Nitride under Visible Light Irradiation. *ACS Catal.* **2015**, *5* (5), 3058–3066. https://doi.org/10.1021/acscatal.5b00408.

(9) Shi, L.; Yang, L.; Zhou, W.; Liu, Y.; Yin, L.; Hai, X.; Song, H.; Ye, J. Photoassisted

Construction of Holey Defective g-C_3N_4 Photocatalysts for Efficient Visible-Light-Driven H_2O_2 Production. *Small* **2018**, *14* (9), 1703142. https://doi.org/10.1002/smll.201703142.

(10) Qu, X.; Hu, S.; Li, P.; Li, Z.; Wang, H.; Ma, H.; Li, W. The Effect of Embedding N Vacancies into g-C_3N_4 on the Photocatalytic H_2O_2 Production Ability via H_2 Plasma Treatment. *Diam. Relat. Mater.* **2018**, *86*, 159–166. https://doi.org/10.1016/j.diamond.2018.04.027.

(11) Wang, R.; Zhang, X.; Li, F.; Cao, D.; Pu, M.; Han, D.; Yang, J.; Xiang, X. Energy-Level Dependent H_2O_2 Production on Metal-Free, Carbon-Content Tunable Carbon Nitride Photocatalysts. *J. Energy Chem.* **2018**, *27* (2), 343–350. https://doi.org/10.1016/j.jechem.2017.12.014.

(12) Zhao, S.; Guo, T.; Li, X.; Xu, T.; Yang, B.; Zhao, X. Carbon Nanotubes Covalent Combined with Graphitic Carbon Nitride for Photocatalytic Hydrogen Peroxide Production under Visible Light. *Appl. Catal. B Environ.* **2018**, *224*, 725–732. https://doi.org/10.1016/j.apcatb.2017.11.005.

(13) Kim, H.; Choi, Y.; Hu, S.; Choi, W.; Kim, J.-H. Photocatalytic Hydrogen Peroxide Production by Anthraquinone-Augmented Polymeric Carbon Nitride. *Appl. Catal. B Environ.* **2018**, *229*, 121–129. https://doi.org/10.1016/j.apcatb.2018.01.060.

(14) Kim, H.; Gim, S.; Jeon, T. H.; Kim, H.; Choi, W. Distorted Carbon Nitride Structure with Substituted Benzene Moieties for Enhanced Visible Light Photocatalytic Activities. *ACS Appl. Mater. Interfaces* **2017**, *9* (46), 40360–40368. https://doi.org/10.1021/acsami.7b14191.

(15) Dong, S.; Liu, C.; Chen, Y. Boosting Exciton Dissociation and Molecular Oxygen Activation by In-Plane Grafting Nitrogen-Doped Carbon Nanosheets to Graphitic Carbon Nitride for Enhanced Photocatalytic Performance. *J. Colloid Interface Sci.* **2019**, *553*, 59–70. https://doi.org/10.1016/j.jcis.2019.06.021.

(16) Bai, J.; Sun, Y.; Li, M.; Yang, L.; Li, J. The Effect of Phosphate Modification on the Photocatalytic H_2O_2 Production Ability of g-C_3N_4 Catalyst Prepared via Acid-Hydrothermal Post-Treatment. *Diam. Relat. Mater.* **2018**, *87*, 1–9. https://doi.org/10.1016/j.diamond.2018.05.004.

(17) Kofuji, Y.; Ohkita, S.; Shiraishi, Y.; Sakamoto, H.; Tanaka, S.; Ichikawa, S.; Hirai, T. Graphitic Carbon Nitride Doped with Biphenyl Diimide: Efficient Photocatalyst for Hydrogen Peroxide Production from Water and Molecular Oxygen by Sunlight. *ACS Catal.* **2016**, *6* (10), 7021–7029. https://doi.org/10.1021/acscatal.6b02367.

(18) Kofuji, Y.; Ohkita, S.; Shiraishi, Y.; Sakamoto, H.; Ichikawa, S.; Tanaka, S.; Hirai, T. Mellitic Triimide-Doped Carbon Nitride as Sunlight-Driven Photocatalysts for Hydrogen Peroxide Production. *ACS Sustain. Chem. Eng.* **2017**, *5* (8), 6478–6485. https://doi.org/10.1021/acssuschemeng.7b00575.

(19) Kofuji, Y.; Isobe, Y.; Shiraishi, Y.; Sakamoto, H.; Tanaka, S.; Ichikawa, S.; Hirai, T. Carbon Nitride–Aromatic Diimide–Graphene Nanohybrids: Metal-Free Photocatalysts for Solar-to-Hydrogen Peroxide Energy Conversion with 0.2% Efficiency. *J. Am. Chem. Soc.* **2016**, *138* (31), 10019–10025. https://doi.org/10.1021/jacs.6b05806.

(20) Kofuji, Y.; Isobe, Y.; Shiraishi, Y.; Sakamoto, H.; Ichikawa, S.; Tanaka, S.; Hirai, T. Hydrogen Peroxide Production on a Carbon Nitride–Boron Nitride-Reduced Graphene Oxide Hybrid Photocatalyst under Visible Light. *ChemCatChem* **2018**, *10* (9), 2070–2077. https://doi.org/10.1002/cctc.201701683.

(21) Zheng, Y.; Yu, Z.; Ou, H.; Asiri, A. M.; Chen, Y.; Wang, X. Black Phosphorus and Polymeric Carbon Nitride Heterostructure for Photoinduced Molecular Oxygen Activation. *Adv. Funct. Mater.* **2018**, *28* (10), 1705407. https://doi.org/10.1002/adfm.201705407.

(22) Yang, Y.; Zhang, C.; Huang, D.; Zeng, G.; Huang, J.; Lai, C.; Zhou, C.; Wang, W.; Guo, H.; Xue, W.; Deng, R.; Cheng, M.; Xiong, W. Boron Nitride Quantum Dots Decorated Ultrathin Porous g-C_3N_4: Intensified Exciton Dissociation and Charge Transfer for Promoting Visible-Light-Driven Molecular Oxygen Activation. *Appl. Catal. B Environ.* **2019**, *245*, 87–99. https://doi.org/10.1016/j.apcatb.2018.12.049.

(23) He, Z.; Kim, C.; Lin, L.; Jeon, T. H.; Lin, S.; Wang, X.; Choi, W. Formation of Heterostructures via Direct Growth CN on H-BN Porous Nanosheets for Metal-Free Photocatalysis. *Nano Energy* **2017**, *42*, 58–68. https://doi.org/10.1016/j.nanoen.2017.10.043.

(24) Moon, G.; Fujitsuka, M.; Kim, S.; Majima, T.; Wang, X.; Choi, W. Eco-Friendly Photochemical Production of H_2O_2 through O_2 Reduction over Carbon Nitride Frameworks Incorporated with Multiple Heteroelements. *ACS Catal.* **2017**, *7* (4), 2886–2895. https://doi.org/10.1021/acscatal.6b03334.

(25) Peng, Y.; Wang, L.; Liu, Y.; Chen, H.; Lei, J.; Zhang, J. Visible-Light-Driven Photocatalytic H_2O_2 Production on g-C_3N_4 Loaded with CoP as a Noble Metal Free Cocatalyst. *Eur. J. Inorg. Chem.* **2017**, *2017* (40), 4797–4802. https://doi.org/10.1002/ejic.201700930.

(26) Chang, X.; Yang, J.; Han, D.; Zhang, B.; Xiang, X.; He, J. Enhancing Light-Driven

Production of Hydrogen Peroxide by Anchoring Au onto C_3N_4 Catalysts. *Catalysts* **2018**, *8* (4), 147. https://doi.org/10.3390/catal8040147.

(27) Hu, S.; Qu, X.; Li, P.; Wang, F.; Li, Q.; Song, L.; Zhao, Y.; Kang, X. Photocatalytic Oxygen Reduction to Hydrogen Peroxide over Copper Doped Graphitic Carbon Nitride Hollow Microsphere: The Effect of Cu(I)-N Active Sites. *Chem. Eng. J.* **2018**, *334*, 410–418. https://doi.org/10.1016/j.cej.2017.10.016.

(28) Kim, S.; Moon, G.; Kim, H.; Mun, Y.; Zhang, P.; Lee, J.; Choi, W. Selective Charge Transfer to Dioxygen on KPF6-Modified Carbon Nitride for Photocatalytic Synthesis of H_2O_2 under Visible Light. *J. Catal.* **2018**, *357*, 51–58. https://doi.org/10.1016/j.jcat.2017.10.002.

(29) Li, X.; Zhang, J.; Zhou, F.; Zhang, H.; Bai, J.; Wang, Y.; Wang, H. Preparation of N-Vacancy-Doped g-C_3N_4 with Outstanding Photocatalytic H_2O_2 Production Ability by Dielectric Barrier Discharge Plasma Treatment. *Chinese J. Catal.* **2018**, *39* (6), 1090–1098. https://doi.org/10.1016/S1872-2067(18)63046-3.

(30) Li, Z.; Xiong, N.; Gu, G. Fabrication of a Full-Spectrum-Response $Cu_2(OH)_2$ CO_3/g-C_3N_4 Heterojunction Catalyst with Outstanding Photocatalytic H_2O_2 Production Performance via a Self-Sacrificial Method. *Dalt. Trans.* **2019**, *48* (1), 182–189. https://doi.org/10.1039/C8DT04081H.

(31) Qu, X.; Hu, S.; Bai, J.; Li, P.; Lu, G.; Kang, X. Synthesis of Band Gap-Tunable Alkali Metal Modified Graphitic Carbon Nitride with Outstanding Photocatalytic H_2O_2 Production Ability via Molten Salt Method. *J. Mater. Sci. Technol.* **2018**, *34* (10), 1932–1938. https://doi.org/10.1016/j.jmst.2018.04.019.

(32) Wei, Z.; Liu, M.; Zhang, Z.; Yao, W.; Tan, H.; Zhu, Y. Efficient Visible-Light-Driven Selective Oxygen Reduction to Hydrogen Peroxide by Oxygen-Enriched Graphitic Carbon Nitride Polymers. *Energy Environ. Sci.* **2018**, *11* (9), 2581–2589. https://doi.org/10.1039/C8EE01316K.

(33) Zhang, C.; Bai, J.; Ma, L.; Lv, Y.; Wang, F.; Zhang, X.; Yuan, X.; Hu, S. Synthesis of Halogen Doped Graphite Carbon Nitride Nanorods with Outstanding Photocatalytic H_2O_2 Production Ability via Saturated NH_4X (X = Cl, Br) Solution-Hydrothermal Post-Treatment. *Diam. Relat. Mater.* **2018**, *87* (April), 215–222. https://doi.org/10.1016/j.diamond.2018.06.013.

(34) Zhao, S.; Zhao, X.; Ouyang, S.; Zhu, Y. Polyoxometalates Covalently Combined with Graphitic Carbon Nitride for Photocatalytic Hydrogen Peroxide Production. *Catal. Sci. Technol.* **2018**, *8* (6), 1686–1695. https://doi.org/10.1039/C8CY00043C.

(35) Tian, J.; Wu, T.; Wang, D.; Pei, Y.; Qiao, M.; Zong, B. One-Pot Synthesis of Potassium and Phosphorus-Doped Carbon Nitride Catalyst Derived from Urea for Highly Efficient Visible Light-Driven Hydrogen Peroxide Production. *Catal. Today* **2019**, *330*, 171–178. https://doi.org/10.1016/j.cattod.2018.07.039.

(36) Zhao, S.; Zhao, X. Insights into the Role of Singlet Oxygen in the Photocatalytic Hydrogen Peroxide Production over Polyoxometalates-Derived Metal Oxides Incorporated into Graphitic Carbon Nitride Framework. *Appl. Catal. B Environ.* **2019**, *250*, 408–418. https://doi.org/10.1016/j.apcatb.2019.02.031.

(37) Zuo, G.; Liu, S.; Wang, L.; Song, H.; Zong, P.; Hou, W.; Li, B.; Guo, Z.; Meng, X.; Du, Y.; Wang, T.; Roy, V. A. L. Finely Dispersed Au Nanoparticles on Graphitic Carbon Nitride as Highly Active Photocatalyst for Hydrogen Peroxide Production. *Catal. Commun.* **2019**, *123*, 69–72. https://doi.org/10.1016/j.catcom.2019.02.011.

(38) Yang, Y.; Zeng, Z.; Zeng, G.; Huang, D.; Xiao, R.; Zhang, C.; Zhou, C.; Xiong, W.; Wang, W.; Cheng, M.; Xue, W.; Guo, H.; Tang, X.; He, D. Ti_3C_2 Mxene/Porous g-C3N4 Interfacial Schottky Junction for Boosting Spatial Charge Separation in Photocatalytic H_2O_2 Production. *Appl. Catal. B Environ.* **2019**, *258*, 117956. https://doi.org/10.1016/j.apcatb.2019.117956.

(39) Zhang, P.; Sun, D.; Cho, A.; Weon, S.; Lee, S.; Lee, J.; Han, J. W.; Kim, D.-P.; Choi, W. Modified Carbon Nitride Nanozyme as Bifunctional Glucose Oxidase-Peroxidase for Metal-Free Bioinspired Cascade Photocatalysis. *Nat. Commun.* **2019**, *10* (1), 940. https://doi.org/10.1038/s41467-019-08731-y.

(40) Moon, G. H.; Kim, W.; Bokare, A. D.; Sung, N. E.; Choi, W. Solar Production of H_2O_2 on Reduced Graphene Oxide-TiO_2 Hybrid Photocatalysts Consisting of Earth-Abundant Elements Only. *Energy Environ. Sci.* **2014**, *7* (12), 4023–4028. https://doi.org/10.1039/c4ee02757d.

(41) Maurino, V.; Minero, C.; Mariella, G.; Pelizzetti, E. Sustained Production of H_2O_2 on Irradiated TiO_2–Fluoride Systems. *Chem. Commun.* **2005**, No. 20, 2627. https://doi.org/10.1039/b418789j.

(42) Teranishi, M.; Naya, S.; Tada, H. In Situ Liquid Phase Synthesis of Hydrogen Peroxide from Molecular Oxygen Using Gold Nanoparticle-Loaded Titanium(IV) Dioxide Photocatalyst. *J. Am. Chem. Soc.* **2010**, *132* (23), 7850–7851. https://doi.org/10.1021/ja102651g.

(43) Tsukamoto, D.; Shiro, A.; Shiraishi, Y.; Sugano, Y.; Ichikawa, S.; Tanaka, S.; Hirai, T. Photocatalytic H_2O_2 Production from Ethanol/O_2 System Using TiO_2 Loaded with Au–

Ag Bimetallic Alloy Nanoparticles. *ACS Catal.* **2012**, *2* (4), 599–603. https://doi.org/10.1021/cs2006873.

(44) Shiraishi, Y.; Kanazawa, S.; Tsukamoto, D.; Shiro, A.; Sugano, Y.; Hirai, T. Selective Hydrogen Peroxide Formation by Titanium Dioxide Photocatalysis with Benzylic Alcohols and Molecular Oxygen in Water. *ACS Catal.* **2013**, *3* (10), 2222–2227. https://doi.org/10.1021/cs400511q.

(45) Teranishi, M.; Naya, S.; Tada, H. Temperature- and pH-Dependence of Hydrogen Peroxide Formation from Molecular Oxygen by Gold Nanoparticle-Loaded Titanium(IV) Oxide Photocatalyst. *J. Phys. Chem. C* **2016**, *120* (2), 1083–1088. https://doi.org/10.1021/acs.jpcc.5b10626.

(46) Zheng, L.; Su, H.; Zhang, J.; Walekar, L. S.; Vafaei Molamahmood, H.; Zhou, B.; Long, M.; Hu, Y. H. Highly Selective Photocatalytic Production of H_2O_2 on Sulfur and Nitrogen Co-Doped Graphene Quantum Dots Tuned TiO_2. *Appl. Catal. B Environ.* **2018**, *239* (August), 475–484. https://doi.org/10.1016/j.apcatb.2018.08.031.

(47) Xiong, X.; Zhang, X.; Liu, S.; Zhao, J.; Xu, Y. Sustained Production of H_2O_2 in Alkaline Water Solution Using Borate and Phosphate-Modified Au/TiO_2 Photocatalysts. *Photochem. Photobiol. Sci.* **2018**, *17* (8), 1018–1022. https://doi.org/10.1039/C8PP00177D.

(48) Baran, T.; Wojtyła, S.; Minguzzi, A.; Rondinini, S.; Vertova, A. Achieving Efficient H_2O_2 Production by a Visible-Light Absorbing, Highly Stable Photosensitized TiO_2. *Appl. Catal. B Environ.* **2019**, *244*, 303–312. https://doi.org/10.1016/j.apcatb.2018.11.044.

(49) Zuo, G.; Li, B.; Guo, Z.; Wang, L.; Yang, F.; Hou, W.; Zhang, S.; Zong, P.; Liu, S.; Meng, X.; Du, Y.; Wang, T.; Roy, V. A. L. Efficient Photocatalytic Hydrogen Peroxide Production over TiO_2 Passivated by SnO_2. *Catalysts* **2019**, *9* (7), 623. https://doi.org/10.3390/catal9070623.

(50) Hirakawa, H.; Shiota, S.; Shiraishi, Y.; Sakamoto, H.; Ichikawa, S.; Hirai, T. Au Nanoparticles Supported on $BiVO_4$: Effective Inorganic Photocatalysts for H_2O_2 Production from Water and O_2 under Visible Light. *ACS Catal.* **2016**, *6* (8), 4976–4982. https://doi.org/10.1021/acscatal.6b01187.

(51) Lu, Y.; Huang, Y.; Zhang, Y.; Huang, T.; Li, H.; Cao, J.; Ho, W. Effects of H_2O_2 Generation over Visible Light-Responsive Bi/$Bi_2O_2{-}CO_3$ Nanosheets on Their Photocatalytic NO Removal Performance. *Chem. Eng. J.* **2019**, *363*, 374–382. https://doi.org/10.1016/j.cej.2019.01.172.

(52) Lee, J. H.; Cho, H.; Park, S. O.; Hwang, J. M.; Hong, Y.; Sharma, P.; Jeon, W. C.; Cho, Y.; Yang, C.; Kwak, S. K.; Moon, H. R.; Jang, J. High Performance H_2O_2 Production Achieved by Sulfur-Doped Carbon on CdS Photocatalyst via Inhibiting Reverse H_2O_2 Decomposition. *Appl. Catal. B Environ.* **2021**, *284,* 119690. https://doi.org/10.1016/j.apcatb.2020.119690.

(53) Kim, H.; Kwon, O. S.; Kim, S.; Choi, W.; Kim, J.-H. Harnessing Low Energy Photons (635 nm) for the Production of H_2O_2 Using Upconversion Nanohybrid Photocatalysts. *Energy Environ. Sci.* **2016**, *9* (3), 1063–1073. https://doi.org/10.1039/C5EE03115J.

(54) Song, H.; Wei, L.; Chen, C.; Wen, C.; Han, F. Photocatalytic Production of H_2O_2 and Its in Situ Utilization over Atomic-Scale Au Modified MoS_2 Nanosheets. *J. Catal.* **2019**, *376*, 198–208. https://doi.org/10.1016/j.jcat.2019.06.015.

(55) Shiraishi, Y.; Takii, T.; Hagi, T.; Mori, S.; Kofuji, Y.; Kitagawa, Y.; Tanaka, S.; Ichikawa, S.; Hirai, T. Resorcinol–Formaldehyde Resins as Metal-Free Semiconductor Photocatalysts for Solar-to-Hydrogen Peroxide Energy Conversion. *Nat. Mater.* **2019**, *18* (9), 985–993. https://doi.org/10.1038/s41563-019-0398-0.

(56) Isaka, Y.; Kawase, Y.; Kuwahara, Y.; Mori, K.; Yamashita, H. Two-Phase System Utilizing Hydrophobic Metal–Organic Frameworks (MOFs) for Photocatalytic Synthesis of Hydrogen Peroxide. *Angew. Chemie - Int. Ed.* **2019**, *58* (16), 5402–5406. https://doi.org/10.1002/anie.201901961.

(57) Isaka, Y.; Kondo, Y.; Kawase, Y.; Kuwahara, Y.; Mori, K.; Yamashita, H. Photocatalytic Production of Hydrogen Peroxide through Selective Two-Electron Reduction of Dioxygen Utilizing Amine-Functionalized MIL-125 Deposited with Nickel Oxide Nanoparticles. *Chem. Commun.* **2018**, *54* (67), 9270–9273. https://doi.org/10.1039/C8CC02679C.

(58) Gogoi, S.; Karak, N. Solar-Driven Hydrogen Peroxide Production Using Polymer-Supported Carbon Dots as Heterogeneous Catalyst. *Nano-Micro Lett.* **2017**, *9* (4), 40. https://doi.org/10.1007/s40820-017-0143-7.

(59) Yamada, Y.; Nomura, A.; Miyahigashi, T.; Ohkubo, K.; Fukuzumi, S. Acetate Induced Enhancement of Photocatalytic Hydrogen Peroxide Production from Oxalic Acid and Dioxygen. *J. Phys. Chem. A* **2013**, *117* (18), 3751–3760. https://doi.org/10.1021/jp312795f.

(60) Zhuang, H.; Yang, L.; Xu, J.; Li, F.; Zhang, Z.; Lin, H.; Long, J.; Wang, X. Robust Photocatalytic H_2O_2 Production by Octahedral $Cd_3(C_3N_3S_3)_2$ Coordination Polymer under Visible Light. *Sci. Rep.* **2015**, *5* (1), 16947. https://doi.org/10.1038/srep16947.

(61) Wang, H.; Yang, C.; Chen, F.; Zheng, G.; Han, Q. A Crystalline Partially Fluorinated Triazine Covalent Organic Framework for Efficient Photosynthesis of Hydrogen Peroxide. *Angew. Chemie Int. Ed.* **2022**, *200438*. https://doi.org/10.1002/anie.202202328.

(62) Tan, F.; Zheng, Y.; Zhou, Z.; Wang, H.; Dong, X.; Yang, J.; Ou, Z.; Qi, H.; Liu, W.; Zheng, Z.; Chen, X. Aqueous Synthesis of Covalent Organic Frameworks as Photocatalysts for Hydrogen Peroxide Production. *CCS Chem.* **2022**, 1–11. https://doi.org/10.31635/ccschem.022.202101578.

(63) Liu, L.; Gao, M. Y.; Yang, H.; Wang, X.; Li, X.; Cooper, A. I. Linear Conjugated Polymers for Solar-Driven Hydrogen Peroxide Production: The Importance of Catalyst Stability. *J. Am. Chem. Soc.* **2021**, *143* (46), 19287–19293. https://doi.org/10.1021/jacs.1c09979.
